# New insights into the evolution and functional divergence of the *CIPK* gene family in *Saccharum*

**DOI:** 10.1186/s12864-020-07264-9

**Published:** 2020-12-07

**Authors:** Weihua Su, Yongjuan Ren, Dongjiao Wang, Long Huang, Xueqin Fu, Hui Ling, Yachun Su, Ning Huang, Hanchen Tang, Liping Xu, Youxiong Que

**Affiliations:** 1 Key Laboratory of Sugarcane Biology and Genetic Breeding, Ministry of Agriculture, Fujian Agriculture and Forestry University, Fuzhou, 350002 China; 2 Key Laboratory of Genetics, Breeding and Multiple Utilization of Crops, Ministry of Education, Fujian Agriculture and Forestry University, Fuzhou, 350002 China; 3 Plant Immunity Center, Fujian Agriculture and Forestry University, Fuzhou, 350002 China

**Keywords:** Sugarcane, *CIPK*, Genome-wide, Evolution, Biotic stress, Abiotic stress

## Abstract

**Background:**

Calcineurin B-like protein (CBL)-interacting protein kinases (CIPKs) are the primary components of calcium sensors, and play crucial roles in plant developmental processes, hormone signaling transduction, and in the response to exogenous stresses.

**Results:**

In this study, 48 *CIPK* genes (*SsCIPKs*) were identified from the genome of *Saccharum spontaneum*. Phylogenetic reconstruction suggested that the *SsCIPK* gene family may have undergone six gene duplication events from the last common ancestor (LCA) of *SsCIPKs*. Whole-genome duplications (WGDs) served as the driving force for the amplification of *SsCIPKs*. The Nonsynonymous to synonymous substitution ratio (Ka/Ks) analysis showed that the duplicated genes were possibly under strong purifying selection pressure. The divergence time of these duplicated genes had an average duplication time of approximately 35.66 Mya, suggesting that these duplication events occurred after the divergence of the monocots and eudicots (165 Mya). The evolution of gene structure analysis showed that the *SsCIPK* family genes may involve intron losses. Ten *ScCIPK* genes were amplified from sugarcane (*Saccharum spp*. hybrids). The results of real-time quantitative polymerase chain reaction (qRT-PCR) demonstrated that these ten *ScCIPK* genes had different expression patterns under abscisic acid (ABA), polyethylene glycol (PEG), and sodium chloride (NaCl) stresses. Prokaryotic expression implied that the recombinant proteins of ScCIPK3, − 15 and − 17 could only slightly enhance growth under salinity stress conditions, but the ScCIPK21 did not. Transient *N. benthamiana* plants overexpressing *ScCIPKs* demonstrated that the *ScCIPK* genes were involved in responding to external stressors through the ethylene synthesis pathway as well as to bacterial infections.

**Conclusions:**

In generally, a comprehensive genome-wide analysis of evolutionary relationship, gene structure, motif composition, and gene duplications of *SsCIPK* family genes were performed in *S. spontaneum*. The functional study of expression patterns in sugarcane and allogenic expressions in *E. coli* and *N. benthamiana* showed that *ScCIPKs* played various roles in response to different stresses. Thus, these results improve our understanding of the evolution of the *CIPK* gene family in sugarcane as well as provide a basis for in-depth functional studies of *CIPK* genes in sugarcane.

**Supplementary Information:**

The online version contains supplementary material available at 10.1186/s12864-020-07264-9.

## Background

Throughout the life cycle, plants are often subjected to various environmental situations, including saline soil damage, drought, extreme temperature, and pathogens. To date, plants have evolved complex physiological and genetic mechanisms to cope with these adverse environmental conditions for their growth and development [1, 2]. For instance, when plants respond and adapt to stresses, many stress-related genes are induced [3–5], and a variety of stress resistance-related functional proteins accumulate [6–10]. Calcium has emerged as a ubiquitous second messenger that is involved in multiple physiological, developmental, and signal transduction pathways [11–13]. In plants, the levels of intracellular calcium are modulated in response to a diverse array of signals, including abiotic stresses, biotic stresses, exogenous stimuli, and perform physiological activities [14–17]. The level of regulation in calcium signaling can be achieved via calcium-binding proteins [18–20]. These sensor proteins recognize specific calcium signatures and relay these signals to downstream responses, such as phosphorylation cascades, which in turn regulate gene expression [19].

CIPKs specifically target CBLs to transduce the perceived calcium signal, which belongs to the Ca^2+^-mediated CBL-CIPK network, and respond to diverse stimuli [21, 22]. CIPKs are also designated as SNF1-related protein kinases 3 (SnRK3), which is a group of SnRK belonging to the Ser/Thr protein kinase superfamily CDPK-SnRKs [23]. CIPKs contain three domains, including an N- terminal kinase domain, variable auto-inhibitory domain, and a C-terminal regulatory domain [24, 25]. The N-terminal kinase domain consists of a putative activation loop between the DFG (Asp, Phe, Gly) and APE (Ala, Pro, Glu) motifs. The C-terminal regulatory domain, which consists of 24-amino acid motif, is designated as the NAF/FISL domain (protein families database accession no. PF03822) [11]. The NAF/FISL domain plays a vital role in mediating interactions with CBLs [26].

With the completion of genome-wide sequencing, a number of genes in multigene families have been identified. Based on the available genomic data, 25 *CIPKs* in *Arabidopsis thaliana* [11], 34 *CIPKs* in *Oryza sativa* [13], 43 *CIPKs* in *Zea mays* [27], and 25 *CIPKs* in *Manihot esculenta* [12] have been identified. As reported, CIPK genes are important in responding to various biotic and abiotic stresses, such as low-temperature, drought, and salt stresses. Luo et al. [28] discovered that the ectopic expression of *BdCIPK31* can enhance low-temperature tolerance in tobacco. *BdCIPK31* also plays a role in regulating plant responses to drought and salt stresses [29]. *ZmCIPK16* is believed to be involved in the CBL-CIPK signaling network that is associated with maize responses to salt stress [30]. The CBL-CIPK signaling pathway also plays an important role in response to environmental stress in plants [31]. The salt overly sensitive (SOS) pathway is the first identified CBL-CIPK signaling pathway, and the CBL-CIPK complex contains CBL4 (SOS3) and CIPK24 (SOS2) [32]. CBL4 interacts with CIPK24 and recruits it to the plasma membrane, where it activates the H^+^/Na^+^ (SOS1) reverse transporter to enhance salt tolerance [33]. By phosphorylating and activating the K^+^ channel (AKT1), AtCIPK23 could directly interact with CBL1 to promote K^+^ uptake under low K^+^ conditions in *A. thaliana* and *O. sativa* [34, 35]. Under salt stress, CIPK21 participates in the regulation of response to osmotic stress in *A. thaliana* by interacting with the vacuolar Ca^2+^ sensors CBL2 and CBL3 [36]. To date, only a few *CIPK* have been studied in sugarcane. *CIPK14* has been shown to play a role in conferring drought tolerance in sugarcane [37]. Farani et al. [38] found that *CIPK8* is not only induced by drought stress but also related to sucrose content.

Sugarcane is the world’s most important sugar crop and an important feedstock for the biofuel industry [39]. Various factors, such as susceptibility to biotic and abiotic stresses, complex genome, narrow genetic base, and poor fertility, restrict sugarcane production [40]. The ancestry of current cultivated sugarcane mainly comprise two taxa: the domesticated sugar-producing species *Saccharum officinarum* and the wild relative *S. spontaneum* [41]. Breeding elite cultivars of sugarcane generally requires several years. Hence, using biotechnologies and genetic engineering may accelerate process and improve the quality of sugarcane cultivar. To empirically address the evolution and function of the *CIPK* gene family, we here analyzed comparative genomics analysis with an emphasis on the functional divergence of the *CIPK* gene family in *Saccharum*. In this study, sequence and evolution analysis of the *SsCIPK* genes were conducted using the available sugarcane genome data [42]. In addition, the expression patterns of the *CIPK* gene family in the presence of abscisic acid (ABA), polyethylene glycol (PEG), and sodium chloride (NaCl) were detected by qRT-PCR. The allogenic expressions of *ScCIPKs* were also explored. Under salinity stress, the growth status of *E. coli* cells expressing ScCIPKs was analyzed. Furthermore, their transient overexpression in *Nicotiana benthamiana* were also investigated. The present study provides new insights into the evolution of the *CIPK* gene family as well as highlight its functional divergence in *Saccharum*.

## Results

### Identification of the *CIPK* gene family in *S. spontaneum*

A total of 93 *CIPK* gene sequences were identified in the *S. spontaneum* genome (Supplementary Table S1). Excluding alleles, 48 *CIPK* genes were detected in the *S. spontaneum* genome. The distribution of these 48 *SsCIPK* genes was uneven on the 20 chromosomes (Supplementary Fig. S1). Most of *SsCIPK* genes were located on the proximal regions or distal ends of chromosomes. Chromosomes 1B, 3C, 4A, 5A, 5C, 6A, 8A, and 8B each contained signal *CIPK* gene. Chromosome 2B had the highest number of *SsCIPK* genes (*N* = 6). However, no *SsCIPK* gene was mapped to Chromosomes 1C, 3D, 4B, 4D, 5B, 5D, 6B, 6C, 6D, 7C, 8C, and 8D. According to the different gene coordinate orders on sugarcane chromosomes, 48 *SsCIPK* genes were named from *SsCIPK1* to *33* [genes that were duplicated [42] were designated the same name followed by the letters “a”, “b”, “c”, “d” and “e”). These SsCIPK proteins were 356–621 amino acid (aa) residues in length. The molecular weight (MW) of the SsCIPKs ranged from 38.72 kDa (SsCIPK9) to 69.04 kDa (SsCIPK12), however, their isoelectric points (*p*I) varied from 5.19 (SsCIPK20) to 9.88 (SsCIPK27b). The subcellular locations, palmitoylation sites, and myristoylation sites have also been predicted in this study. Twenty-eight of 48 SsCIPKs were predicted to be located in the chloroplast, indicating that these SsCIPKs may take part in maintaining Ca^2+^ homeostasis in the chloroplast (Table 1). Twenty-two SsCIPKs, including SsCIPK2a, 2b, 3, 4c, 4e, 6, 8a, 8b, 10, 11, 12, 13, 14, 15, 16, 19, 22a, 22b, 23a, 30, 31a, and 33 have palmitoylation sites (Supplementary Table S2). Seventeen CIPKs, including SsCIPK2a, 4b, 4d, 4e, 8b, 10, 17, 18, 19, 20, 22b, 25a, 25b, 28, 30, 32, and 33 have myristoylation sites (Supplementary Table S2).

**Table 1:** Physicochemical properties of *SsCIPK* genes

Gene name	Genome ID	AA size	MW (kDa)	*p*I	Predicted location^a^
*SsCIPK1*	Sspon.01G0001600-1A	458	51.43	8.00	chlo
*SsCIPK2a*	Sspon.01G0008500-1A	409	46.21	7.60	chlo
*SsCIPK2b*	Sspon.01G0008500-1P	405	44.73	5.19	chlo
*SsCIPK3*	Sspon.01G0009190-1A	482	54.95	9.21	chlo
*SsCIPK4a*	Sspon.01G0023830-1A	363	39.42	9.19	cyto
*SsCIPK4b*	Sspon.01G0023830-1P	433	47.33	8.83	cyto
*SsCIPK4c*	Sspon.01G0023830-2P	411	44.72	9.43	E.R.
*SsCIPK4d*	Sspon.01G0023830-3P	433	47.12	9.09	cyto
*SsCIPK4e*	Sspon.01G0023830-4P	432	58.24	8.89	E.R.
*SsCIPK5*	Sspon.01G0034400-1B	421	47.28	7.62	chlo
*SsCIPK6*	Sspon.01G0009200-3D	575	64.14	8.77	plas
*SsCIPK7a*	Sspon.02G0001070-1A	432	46.49	7.68	chlo
*SsCIPK7b*	Sspon.02G0001070-1T	434	46.64	7.71	chlo
*SsCIPK8a*	Sspon.02G0024240-1A	562	63.13	9.45	chlo
*SsCIPK8b*	Sspon.02G0024240-1P	449	50.67	9.22	chlo
*SsCIPK9*	Sspon.02G0030610-1A	356	38.72	9.44	cyto
*SsCIPK10*	Sspon.02G0033090-1B	438	49.85	9.16	chlo
*SsCIPK11*	Sspon.02G0037890-1B	469	51.29	8.95	E.R.
*SsCIPK12*	Sspon.02G0042500-1B	621	69.04	9.54	chlo
*SsCIPK13*	Sspon.02G0044920-1B	403	43.98	9.41	E.R.
*SsCIPK14*	Sspon.02G0000740-2C	448	50.60	7.13	chlo
*SsCIPK15*	Sspon.03G0003630-1A	581	65.21	6.94	chlo
*SsCIPK16*	Sspon.03G0006080-1A	512	56.30	9.02	chlo
*SsCIPK17*	Sspon.03G0015890-1A	463	51.96	6.38	nucl
*SsCIPK18*	Sspon.03G0023620-1A	517	57.18	8.36	chlo
*SsCIPK19*	Sspon.03G0028160-1B	440	48.73	8.56	mito
*SsCIPK20*	Sspon.03G0013670-2B	405	45.24	5.30	cyto
*SsCIPK21*	Sspon.03G0023630-3C	495	55.00	9.25	chlo
*SsCIPK22a*	Sspon.04G0017790-1A	574	64.49	9.02	chlo
*SsCIPK22b*	Sspon.04G0017790-1P	556	62.37	9.08	chlo
*SsCIPK22c*	Sspon.04G0017790-2P	423	48.12	9.10	chlo
*SsCIPK23a*	Sspon.05G0020210-1A	615	68.91	5.34	plas
*SsCIPK23b*	Sspon.05G0020210-1P	434	49.77	8.54	chlo
*SsCIPK23c*	Sspon.05G0020210-2P	400	45.84	8.11	chlo
*SsCIPK24*	Sspon.05G0036030-1C	440	50.40	7.15	chlo
*SsCIPK25a*	Sspon.06G0005650-1A	451	48.86	9.09	plas
*SsCIPK25b*	Sspon.06G0005650-1P	447	48.32	8.99	plas
*SsCIPK26*	Sspon.07G0004410-1A	567	64.05	8.67	chlo
*SsCIPK27a*	Sspon.07G0010390-1A	440	48.24	8.35	nucl
*SsCIPK27b*	Sspon.07G0010390-1P	414	44.63	9.88	nucl
*SsCIPK28*	Sspon.07G0013900-1A	437	49.32	6.55	chlo
*SsCIPK29a*	Sspon.07G0022730-1B	478	53.70	8.66	chlo
*SsCIPK29b*	Sspon.07G0022730-1P	505	56.71	7.71	chlo
*SsCIPK30*	Sspon.07G0026860-1B	459	51.64	6.48	chlo
*SsCIPK31a*	Sspon.05G0021220-2B	482	54.75	9.22	plas
*SsCIPK31b*	Sspon.05G0021220-2P	445	50.49	9.36	chlo
*SsCIPK32*	Sspon.08G0006260-1A	397	45.07	9.30	cyto
*SsCIPK33*	Sspon.08G0020630-1B	481	53.19	8.86	nucl

Legends: *AA* Amino acid, *MW* Molecular weight, *pI* Isoelectric point. ^a^*chlo* Chloroplast, *E.R*. Endoplasmic reticulum, *mito* Mitochondria, *plas* Plasma membrane, *cyto* Cytoplasmic, *nucl* Nuclear

### Motif composition and gene structure of *CIPK* gene family in *S. spontaneum*

**Fig. 1:**
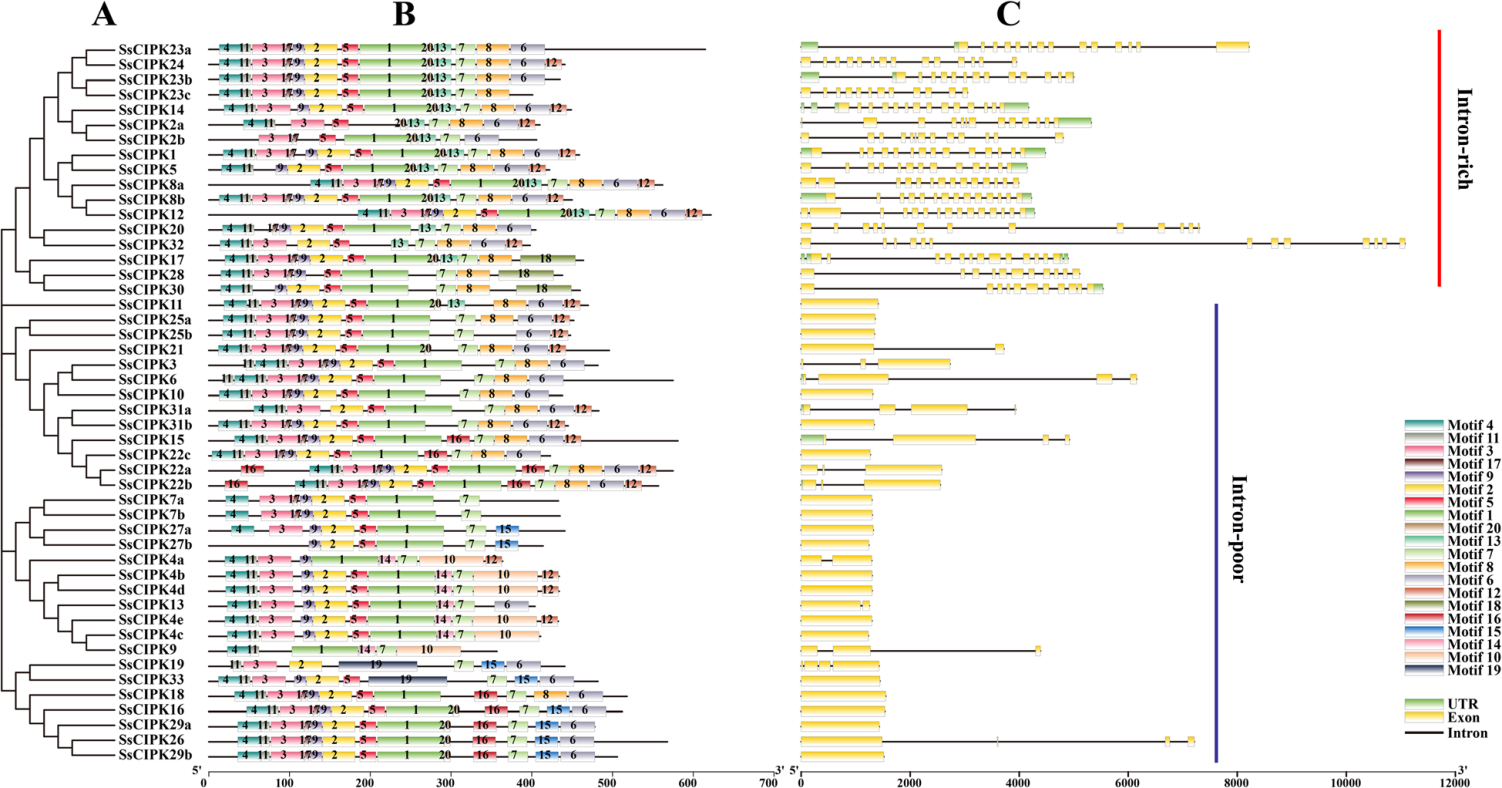
*SsCIPK* phylogenetic relationship, conserved protein motifs, and gene structures. **A** Phylogenetic tree of 48 SsCIPK proteins. The unrooted neighbor-joining phylogenetic tree was constructed using MEGA X. **B** Distributions of conserved motifs in SsCIPK proteins. For motif details refer to Supplementary Fig. S2. **C** Exon/intron organization of the *SsCIPK* genes

To investigate the structural features of *CIPK* genes and their encoded proteins in *S. spontaneum,* the conserved motifs and intron/exon organization were analyzed (Fig. 1 and Supplementary Fig. S2**)**. Figure 1B showed that 20 motifs were identified in SsCIPK proteins. Motif 2 contained the DFG residues and motif 5 had APE residues. Usually, a conserved kinase domain with a putative activation loop in N-terminal of CIPK proteins appeared between the DFG and APE residues. Motif 7 can be annotated as NAF/FISL motif in this study. As shown in Fig. 1, motif 7 was widely distributed in all of the SsCIPK proteins, except for SsCIPK11. SsCIPK2b, 4a, 4b, 4c, 4d, 4e, 7a, 7b, 9, 13, 16, 19, 25b, 26, 27a, 27b, 29a, 29b, and 33 appeared to lost motif 8, which was annotated as a protein-phosphatase interaction (PPI) domain. Some motifs have been found to be unique to several SsCIPKs. For example, motif 18 was specific to SsCIPK17, 28, and 30, but motif 19 was unique to SsCIPK19 and SsCIPK33. Interestingly, SsCIPK22a and SsCIPK22b contained two motif 16.

The exon-intron organization of all of these identified *SsCIPK* genes were examined to gain more insights into the evolution of the CIPK family in sugarcane. As indicated in Fig. 1C, the number of introns in *SsCIPK* genes varied from 0 to 15, and among the 48 *SsCIPK* genes, 31 *SsCIPK* genes were intron-poor with < 4 introns (19 out of 31 without introns), whereas the other 17 *SsCIPK* genes were intron-rich with > 10 introns.

### Phylogenetic analysis of CIPK proteins from *S. spontaneum*, one green algae and six other angiospermaes

A phylogenetic tree consisting of 209 CIPK proteins from *S. spontaneum*, one green algae and six other angiospermaes was constructed using the Neighbor-Joining method (NJ) method to investigate the evolution of *CIPK* orthologs in different plant species. The eight representative species included one green algae (*Chlorella variabilis*) [43], three dicots (*A. thaliana* [11], *Vitis vinifera* [44] and *Populus* [45]) and four monocots (*O. sativa* [11], *Z. mays* [27], *Sorghum bicolor* [44] and *S. spontaneum*) (Supplementary Table S3).

**Fig. 2:**
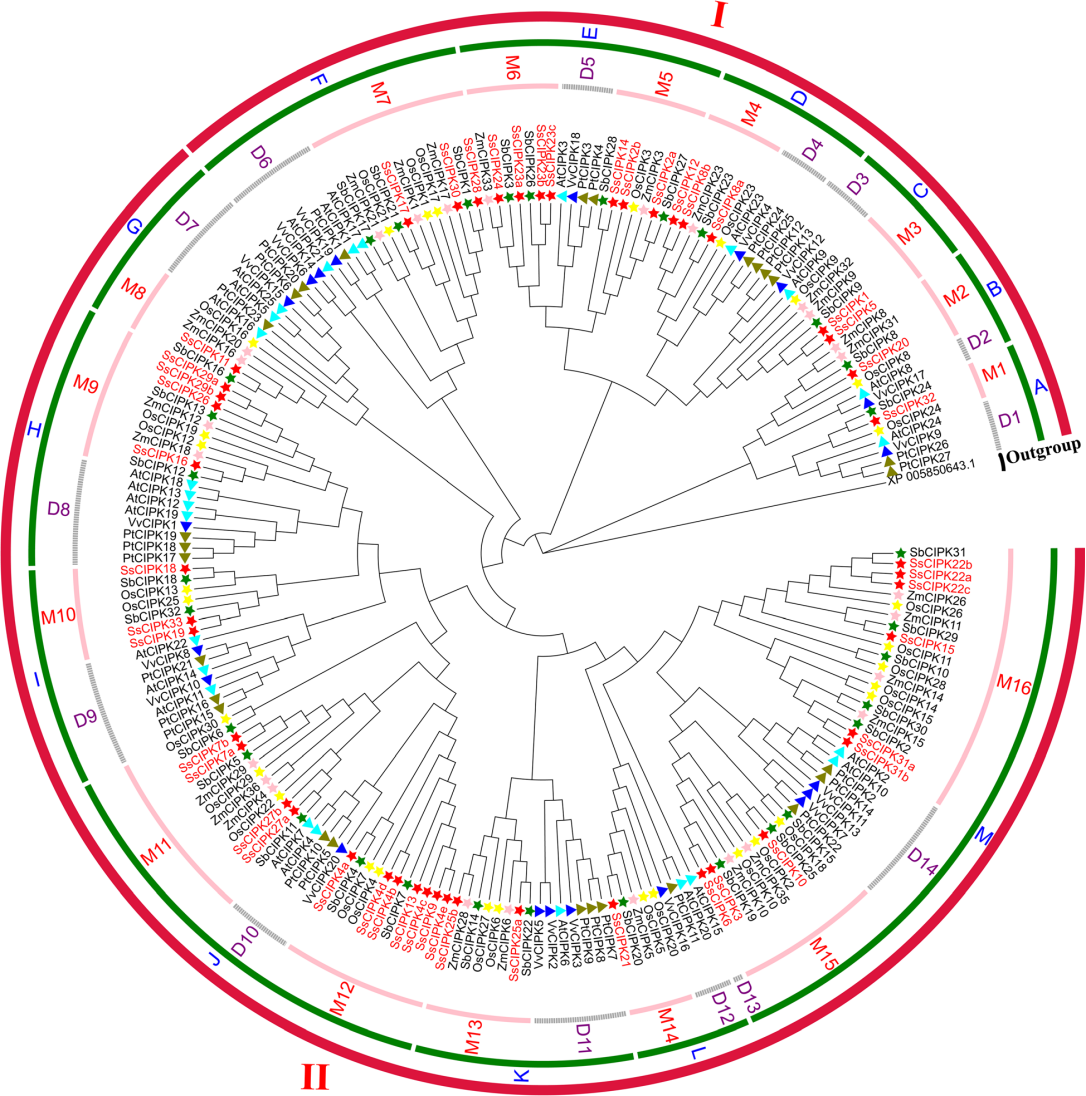
Phylogenetic tree of CIPK proteins from seven plant species. The I and II indicate different groups. The A to M represent different subgroups. The pink arcs and gray dashed represent different subfamilies. The aqua, blue and olive triangles signify *A. thaliana*, *V. vinifera* and *Populus* CIPK proteins, respectively. The yellow, pink, green and red stars represent *O. sativa*, *Z. mays*, *S. bicolor* and *S. spontaneum* CIPK proteins, respectively. *S.spontaneum* CIPK proteins are shown in red. The *CIPK* of *C. variabilis* (GenBank Acc No. XP_005850643.1) as outgroup

As shown in Fig. 2, these angiosperms CIPKs were divided into two major groups (I and II), which could be further classified into 13 subgroups (A - M). The subgroups included 14 dicot subfamilies and 16 monocot subfamilies. Base on the results of Fig. 1, the *SsCIPKs* in group I were intron-rich and the *SsCIPKs* in group II were intron-poor. Each of the subgroups contained CIPKs from both dicots and monocots, suggesting that they had the last common ancestor (LCA) before the monocot-dicot split. In subgroup E, J, and M, CIPKs were distributed into three subfamilies, which could be further assorted into two kinds of subgroups with one consisting of monocot specific genes, and the other containing both dicot and monocot genes, suggesting that gene expansion occurred in monocot species before the divergence of dicots and monocots. The dicot subfamilies generally contained CIPKs from the three examined dicot species, except for D2 and D13. In monocot subfamilies, only M1, M6, M10 and M12 did not contain CIPKs from the four examined monocot species. These four monocot species were all Gramineae. Hence, we speculated that the progenitors of these *CIPK* genes in 12 subfamilies (M2–5, M7–9, M11, and M13–16) may have already existed prior to the divergence of Gramineae.

### Divergence and duplication of the *CIPK* genes in *S. spontaneum*

**Fig. 3:**
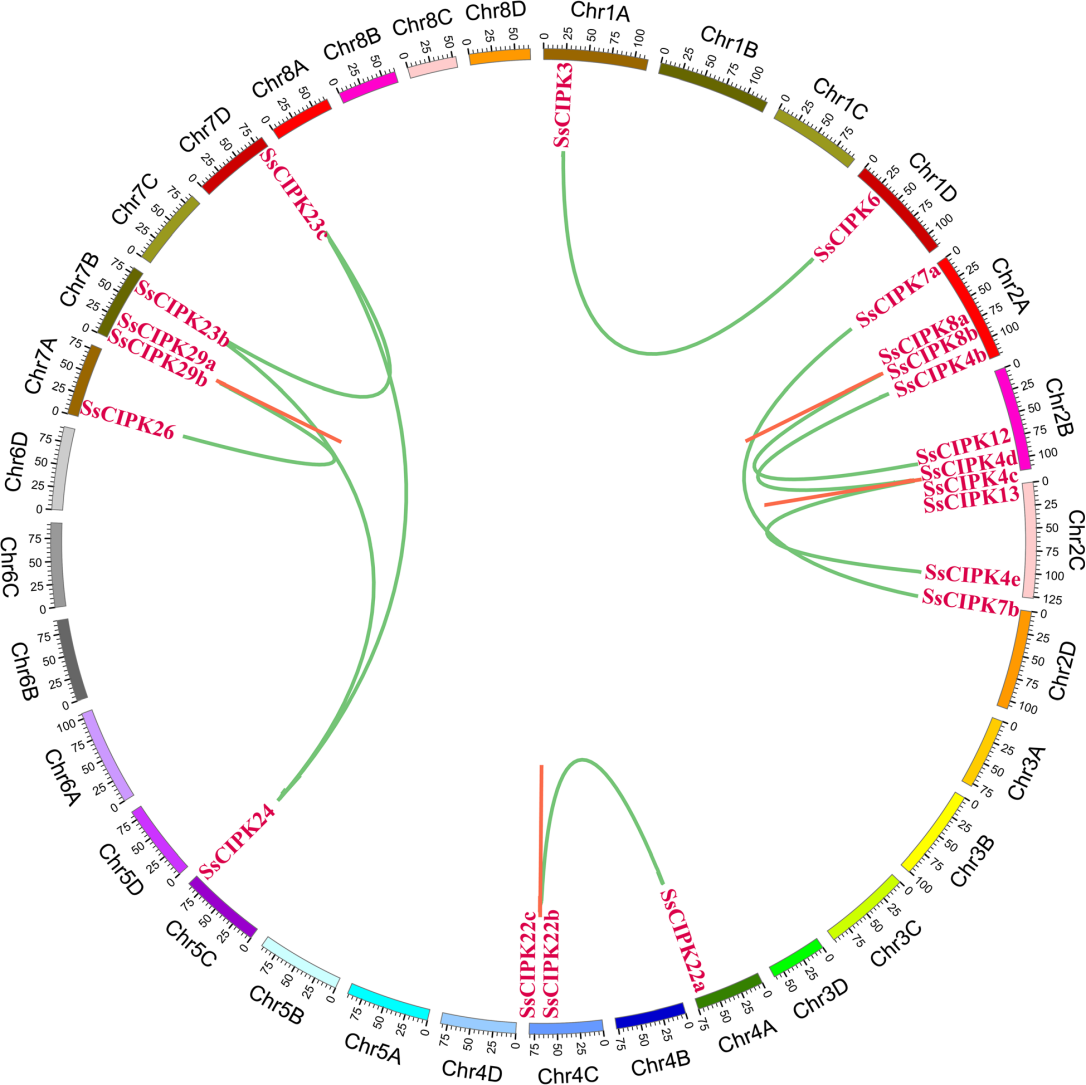
Schematic representations for the chromosomal distribution and interchromosomal relationship of *SsCIPK* duplicated genes. The red lines indicate tandem duplicated *SsCIPK* gene pairs. The chromosome number is indicated at the outer ring of each chromosome

After analyzing the duplication events of *SsCIPK* genes, 16 pairs of *SsCIPKs* were found (Fig. 3). On the basis of defined criteria, five pairs of *SsCIPK* genes (*SsCIPK4d*/*SsCIPK4c*, *SsCIPK4d*/*SsCIPK13*, *SsCIPK8a*/*SsCIPK8b*, *SsCIPK22a*/*SsCIPK22b* and *SsCIPK29a*/*SsCIPK29b*) which linked to each other by red lines were confirmed to be tandem duplicated genes. In addition, the other 11 pairs of *SsCIPK* genes were linked to each other by green lines.

Nonsynonymous to synonymous substitution ratio (Ka/Ks) was analyzed to investigate the duplication of *SsCIPK* genes in *S. spontaneum*, and 16 pairs of paralogous *SsCIPK* genes were calculated (Table 2). The divergence times among the 16 pairs of paralogous *SsCIPK* genes were based on the pairwise synonymous substitution rates (Ks). The results showed that, except for *SsCIPK8a*/*SsCIPK12*, the Ka/Ks ratios of other 15 gene pairs were < 1, suggesting that purifying selection was the main force for driving the gene duplication. Base on divergence time, the gene duplications of *SsCIPK3*/*SsCIPK6*, *SsCIPK8a*/*SsCIPK8b*, *SsCIPK8a*/*SsCIPK12*, *SsCIPK22a*/*SsCIPK22c*, and *SsCIPK26*/*SsCIPK29a* were ancient and divergent. However, the other 11 pairs of *SsCIPKs* underwent recent gene duplications in *Saccharum*.

**Table 2:** Divergence between paralogous *SsCIPK* gene pairs in *S. spontaneum*

Gene Name	Gene Name	Ka	Ks	Ka/Ks	Divergence time (Mya)	*p*-Value (Fisher)
*SsCIPK3*	*SsCIPK6*	0.944043	1.26298	0.747473	103.522951	7.42E-12
*SsCIPK4b*	*SsCIPK4d*	0.00800882	0.0448769	0.178462	3.67843443	0.000203751
*SsCIPK4d*	*SsCIPK4c*	0.0240418	0.232162	0.103556	19.0296721	1.38E-20
*SsCIPK4d*	*SsCIPK4e*	0.032968	0.217475	0.151594	17.8258197	4.34E-17
*SsCIPK4d*	*SsCIPK13*	0.00382849	0.251149	0.0741566	20.5859836	3.99E-23
*SsCIPK7a*	*SsCIPK7b*	0.00358022	0.0622356	0.0575269	5.10127869	9.31E-08
*SsCIPK8a*	*SsCIPK8b*	0.98452	1.05053	0.937162	86.1090164	0.0760462
*SsCIPK8a*	*SsCIPK12*	0.0760462	0.953689	1.06631	78.1712295	0.059028
*SsCIPK22a*	*SsCIPK22b*	0.0191009	0.0471568	0.405051	3.86531148	0.00382849
*SsCIPK22c*	*SsCIPK22b*	0.989297	1.03753	0.953515	85.0434426	0.226151
*SsCIPK23b*	*SsCIPK23c*	0.0141958	0.0266145	0.533387	2.18151639	0.0965334
*SsCIPK24*	*SsCIPK23b*	0.0486279	0.337887	0.143918	27.6956557	8.30E-32
*SsCIPK24*	*SsCIPK23C*	0.0368104	0.349504	0.105322	28.6478689	1.01E-33
*SsCIPK26*	*SsCIPK29a*	0.984641	1.06169	0.927425	87.0237705	0.0916113
*SsCIPK26*	*SsCIPK29b*	0.00598476	0.0256282	0.233522	2.10067213	0.00148432
*SsCIPK29a*	*SsCIPK29b*	2.82E-07	5.29E-07	0.532591	4.3396E-05	0

### Cloning and identification of *CIPK* genes in *Saccharum spp.* hybrid (ROC22)

Through RT-PCR, 10 *CIPK* genes were successfully isolated from *Saccharum* spp. hybrid (ROC22). Phylogenetic tree analysis (Supplementary Fig. S3) and amino acid sequence comparison of CIPKs (Supplementary Table S4) between ROC22 and *S. spontaneum* identified the 10 *CIPK* genes in ROC22, which were designated as *ScCIPK1*, *− 2*, *− 3*, *− 4*, *− 15*, *− 17*, − *20*, *− 21*, − *28*, and − *31*. Table 3 showed that the 10 *ScCIPK* genes encoded polypeptides of 369 (ScCIPK17) to 513 (ScCIPK15) amino acids. The MW of the ScCIPK proteins varied from 41.58 (ScCIPK17) to 57.90 (ScCIPK15) kDa. The *p*I of seven ScCIPKs (ScCIPK1, − 2, − 3, − 4, − 15, − 21, and − 31) were acidic proteins while ScCIPK17, ScCIPK20 and ScCIPK28 were basic protein. The predictions of palmitoylation and myristoylation sites showed that only ScCIPK2 had palmitoylation sites and in the N-terminal domain, both ScCIPK17 and ScCIPK28 had a myristoylation site. Besides, ScCIPK15 had two myristoylation sites, while ScCIPK20 had four myristoylation sites.

**Table 3:** Features of *CIPK* genes in ROC22

Gene name	GenBank Acc No.	AA size	MW (kDa)	*p*I	Predicted location^a^	Palmitoylation sites AA (location)	Myristoylation sites AA (location)
*ScCIPK1*	KX013381	445	50.11	7.62	chlo	–	–
*ScCIPK2*	KX013387	449	50.91	7.99	chlo	C (71, 241)	–
*ScCIPK3*	KX013382	438	50.04	9.27	chlo	–	–
*ScCIPK4*	KX013378	431	46.61	8.18	E.R.	–	–
*ScCIPK15*	KX013384	513	57.90	7.58	chlo	–	G (3, 10),
*ScCIPK17*	KX013386	369	41.58	5.31	chlo	–	G (8)
*ScCIPK20*	KX013380	451	50.92	6.13	cyto	–	G (3, 4, 5, 6)
*ScCIPK21*	KX013379	455	51.11	9.27	chlo	–	–
*ScCIPK28*	KX013385	460	51.81	6.33	chlo	–	G (5)
*ScCIPK31*	KX013383	445	50.48	9.25	chlo	–	–

Legends: *AA* Amino acids, *MW* Molecular weight, *pI* Isoelectric point, ^a^*chlo* Chloroplast, *E.R*. Endoplasmic reticulum, *mito* Mitochondria, *plas* Plasma membrane, *cyto* Cytoplastmic, *nucl* Nuclear, G represents the glycine residue, and C represents the cysteine residue

### Sequence analysis of ten cloned ScCIPK proteins

**Fig. 4:**
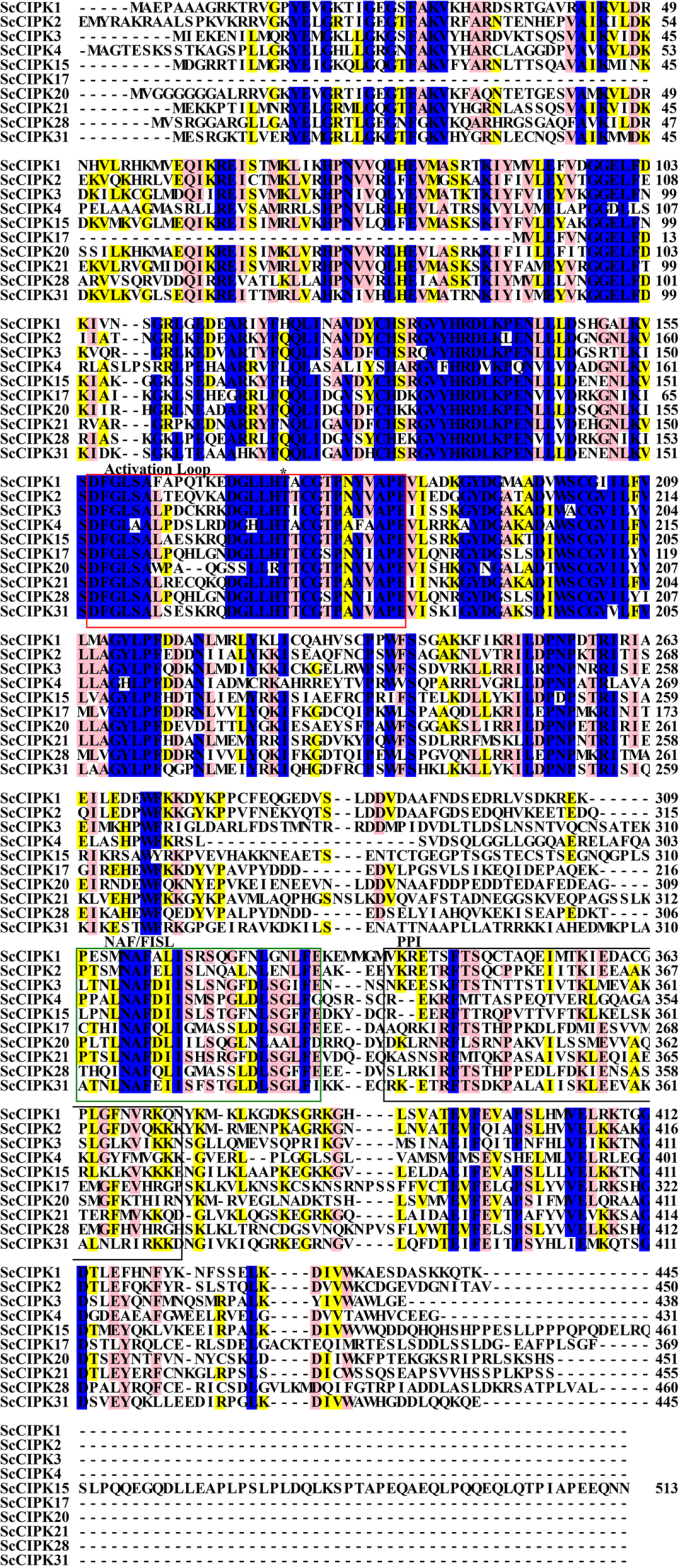
The amino acid alignment of ScCIPK proteins. The red rectangle indicates the activation loop, and the Thr residue is marked by an asterisk. The green rectangle indicates the NAF/FISL motif. The black rectangle indicates the PPI motif. Yellow shading, 50–60% identity; pink shading, 70-80% identity; blue shading, 90-100% identity

DNAMAN 9 program was used to compare the amino acid sequences of 10 cloned ScCIPKs (Fig. 4). The activation loop between DFG and APE motifs and the Thr residue, may be phosphorylated by an upstream protein kinase [24]. The amino acid residues at the 5th, 6th, 7th, 10th, 18th, and 22nd sites of the NAF/FISL motif were completely conserved in the C-terminal regulatory domain. The NAF domain is a conserved CBL interaction module, which has been shown to mediate the interaction with all of the known AtCBL proteins [26]. The PPI motif is necessary and sufficient for the interaction with abscisic acid-insensitive 2 (ABI2) [46]. The fifth amino acid residue of the PPI motif in ScCIPK1 and ScCIPK3 was S (serine) and K (lysine), respectively. Whether the amino acid changes will affect the function of this module still needs to be further verified.

### Expression of *ScCIPK* genes in response to ABA, PEG, and NaCl stress

**Fig. 5:**
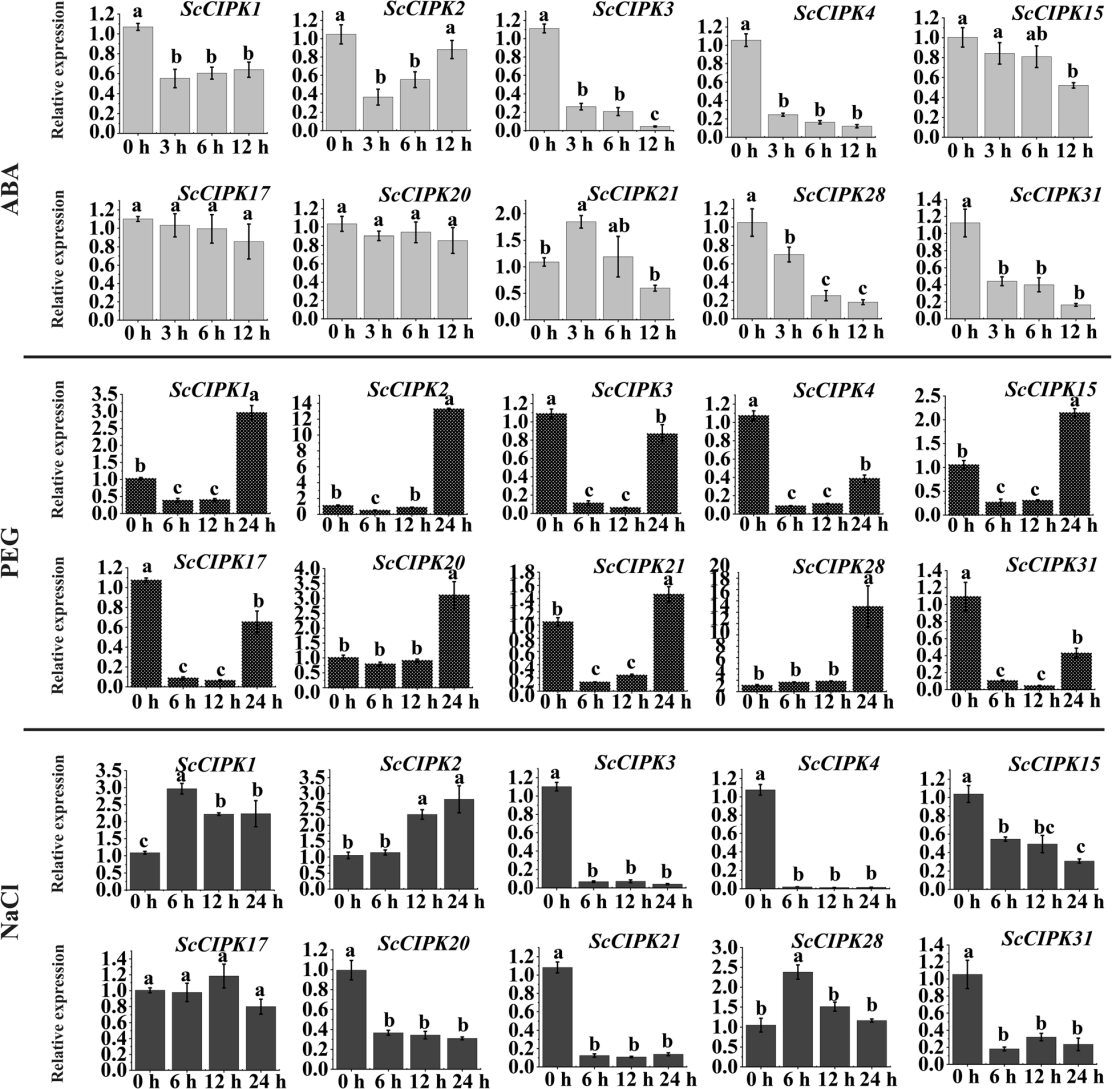
Expression analysis of *ScCIPK* genes in ROC22 plantlets after treatment with 100 μM ABA, 20% PEG, 250 mM NaCl by qRT-PCR. The expression levels of Cullin (*CUL*) and Clathrin adaptor complex (*CAC*) were used for normalization. All data points showed were mean ± SE (*n* = 3). Different lowercase letters indicate a significant difference, as determined by the Duncan’s new multiple range test (*p*-value < 0.05)

The expression of *ScCIPK* genes under ABA, PEG and NaCl stress was detected by qRT-PCR (Fig. 5). Under ABA stress, only the expression of *ScCIPK21* was upregulated at 3 h and 6 h. The mRNA expression of *ScCIPK1, − 2, − 3*, − *4*, *− 15*, *− 28,* and *− 31* was inhibited while that of *ScCIPK17* and *ScCIPK20* did not change. PEG treatment, resulted in the upregulation of *ScCIPK1*, *− 2, − 15*, *− 20*, − *21*, and *− 28* at 24 h, whereas the other four (*﻿ScCIPK3*, − *4*, *− 17*, and *− 31*) were downregulated. In response to NaCl stress, *ScCIPK1, − 2*, and *− 28* was upregulated, whereas *ScCIPK3, − 4, − 15*, *− 20*, *− 21*, and *− 31* were downregulated, and only *ScCIPK17* showed no significant difference between treatment and control.

### Overexpression of *ScCIPKs* in *E. coli* cells under salinity stress

**Fig. 6:**
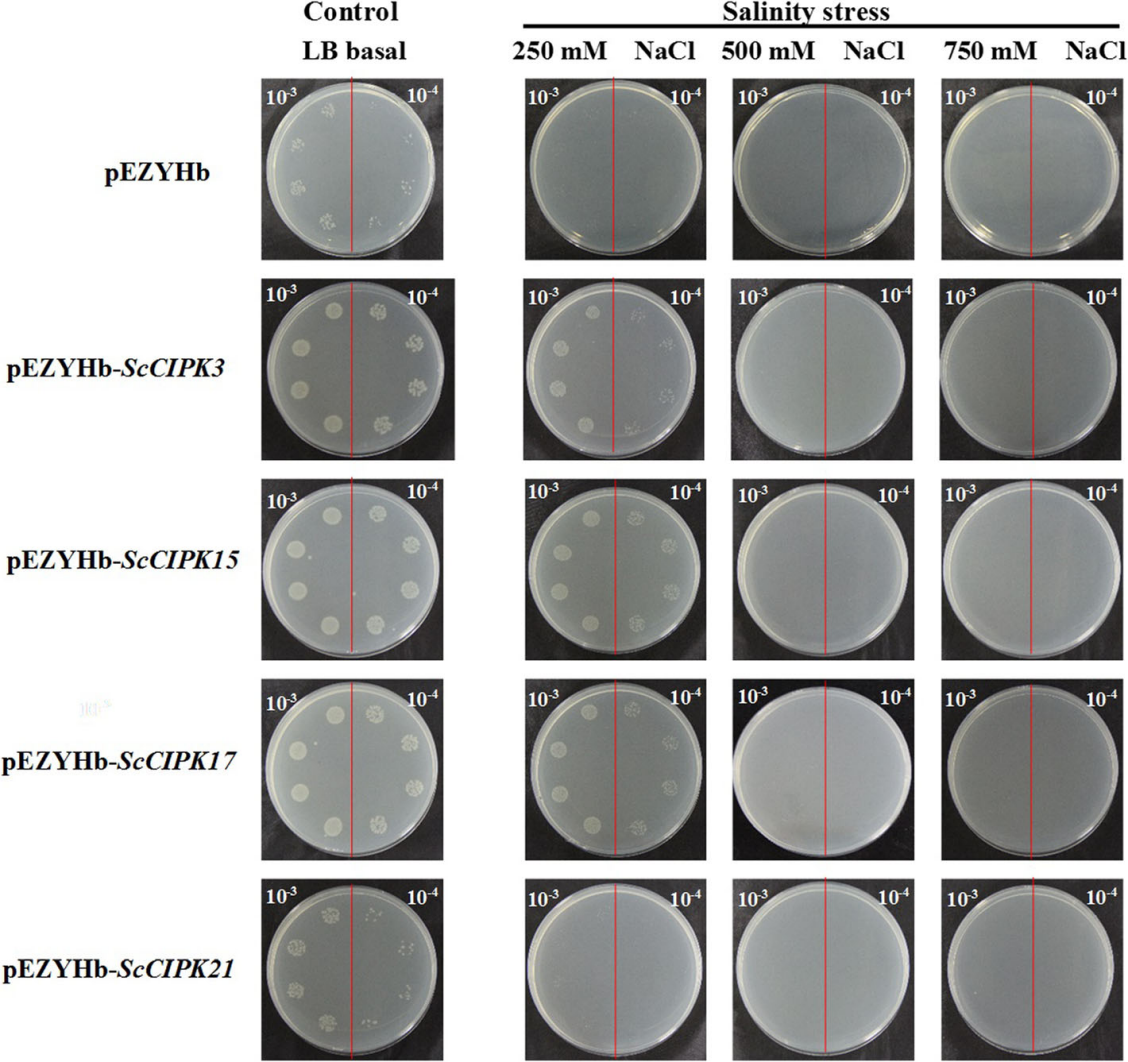
Spot assays for monitoring the growth performance of BL21/pEZYHb and BL21/ pEZYHb-*ScCIPKs* cells. To monitoring the growth performance of BL21/pEZYHb and BL21/ pEZYHb-*ScCIPKs* cells, LB plates without any supplement were used as control. To explore the tolerance of BL21/pEZYHb and BL21/ pEZYHb-*ScCIPKs* cells under salt stress, LB plates were supplemented with NaCl (sodium chloride) (250 mM, 500 mM, and 750 mM)

The growth performance of *E. coli* cells transformed pEZYHb (empty vector) or pEZYHb-*ScCIPKs* (recombinant plasmid) under non-stress (i.e. control) and stress conditions (i.e. high salinity) was tested (Fig. 6). In the spot assay, pEZYHb and pEZYHb-*ScCIPK*-transformed bacterial cells showed a normal growth on solid LB medium (control) with no significant difference. Under salinity stress, either pEZYHb or pEZYHb-*ScCIPK-*transformed bacterial cells all could not grow in the LB plates supplemented with 500 or 750 mM NaCl. These results showed that excessive salt concentration can halt the development of *E. coli* cells. For low-salinity stress, 250 mM NaCl was used, and pEZYHb-*ScCIPK3*-, − *15*- and − *17*-transformed bacterial cultures showed an increase in number with better survival compared to the untransformed cells. These findings suggest that the *E. coli* cells harboring pEZYHb-*ScCIPK3*, − *15* and − *17* enhance the tolerance of bacterial cells under low-salinity stress (250 mM NaCl).

### Transient overexpression of *ScCIPKs* in *N. benthamiana* leaves

**Fig. 7:**
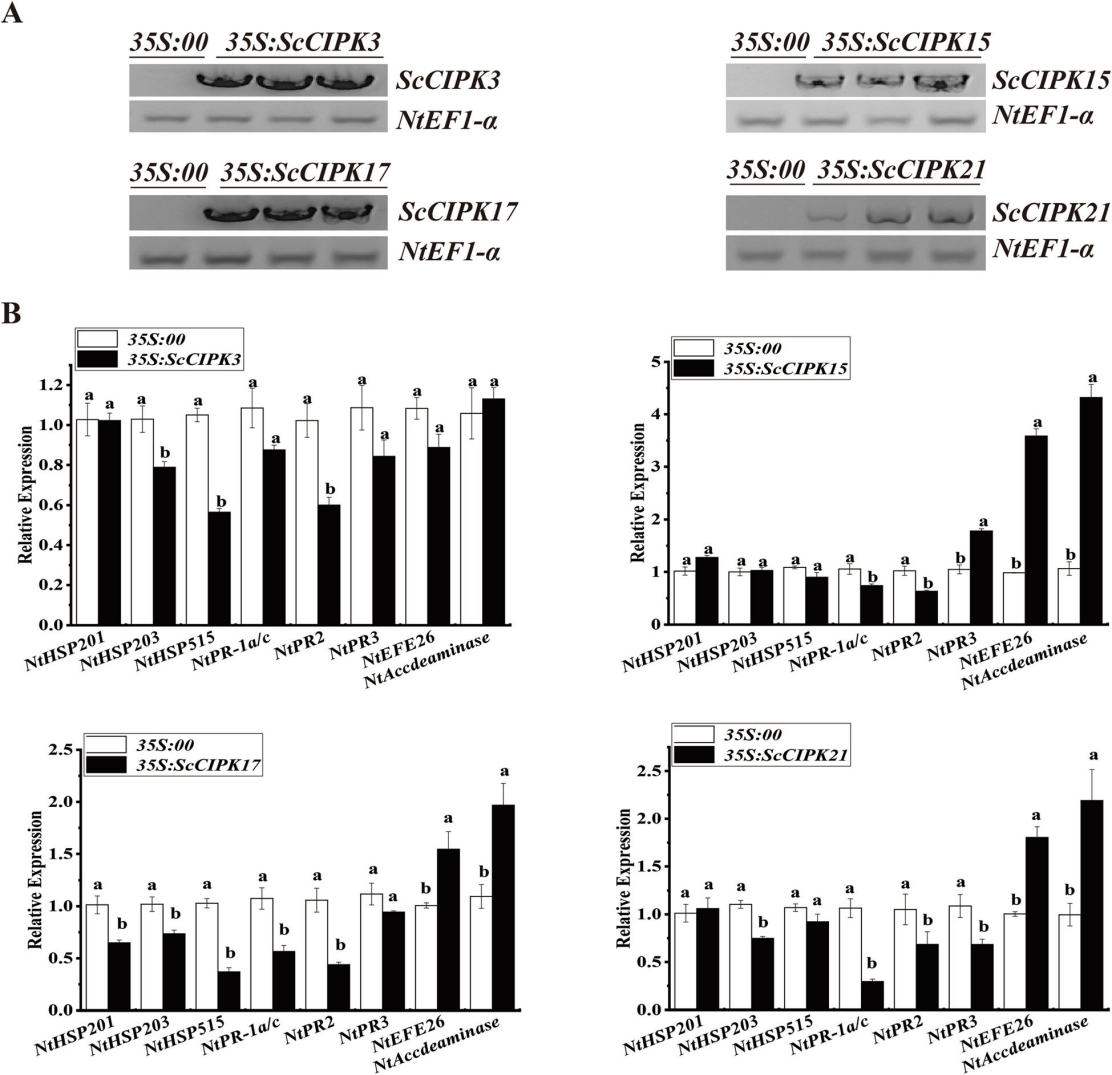
Transient overexpression of *ScCIPKs* in *Nicotiana benthamiana* leaves. **A** RT-PCR analysis of *ScCIPKs* in the *N. benthamiana* leaves 2 days after infiltration with *Agrobacterium* strain GV3101 that carried the vector *35S::00* or *35S::ScCIPKs* (The gels were selected from the same gel with the same exposure, and the unmodified figure was shown in the Supplementary Fig. S4-S7)*.* **B** The transcripts of the immunity-associated marker genes in *35S::ScCIPKs* -transiently expressing leaves at 2 days after infiltration. *NtEF1-α* was used for normalization of the transcript levels. Mock, the *Agrobacterium* strain carrying *35S*::*00*. All of the data points were expressed as the mean ± SE (*n* = 3). Different lowercase letters indicate a significant difference between *35S*::*00* and *35S*::*ScCIPKs* in each gene, as determined by the Duncan’s new multiple range test (*p***-**value < 0.05)

**Fig. 8:**
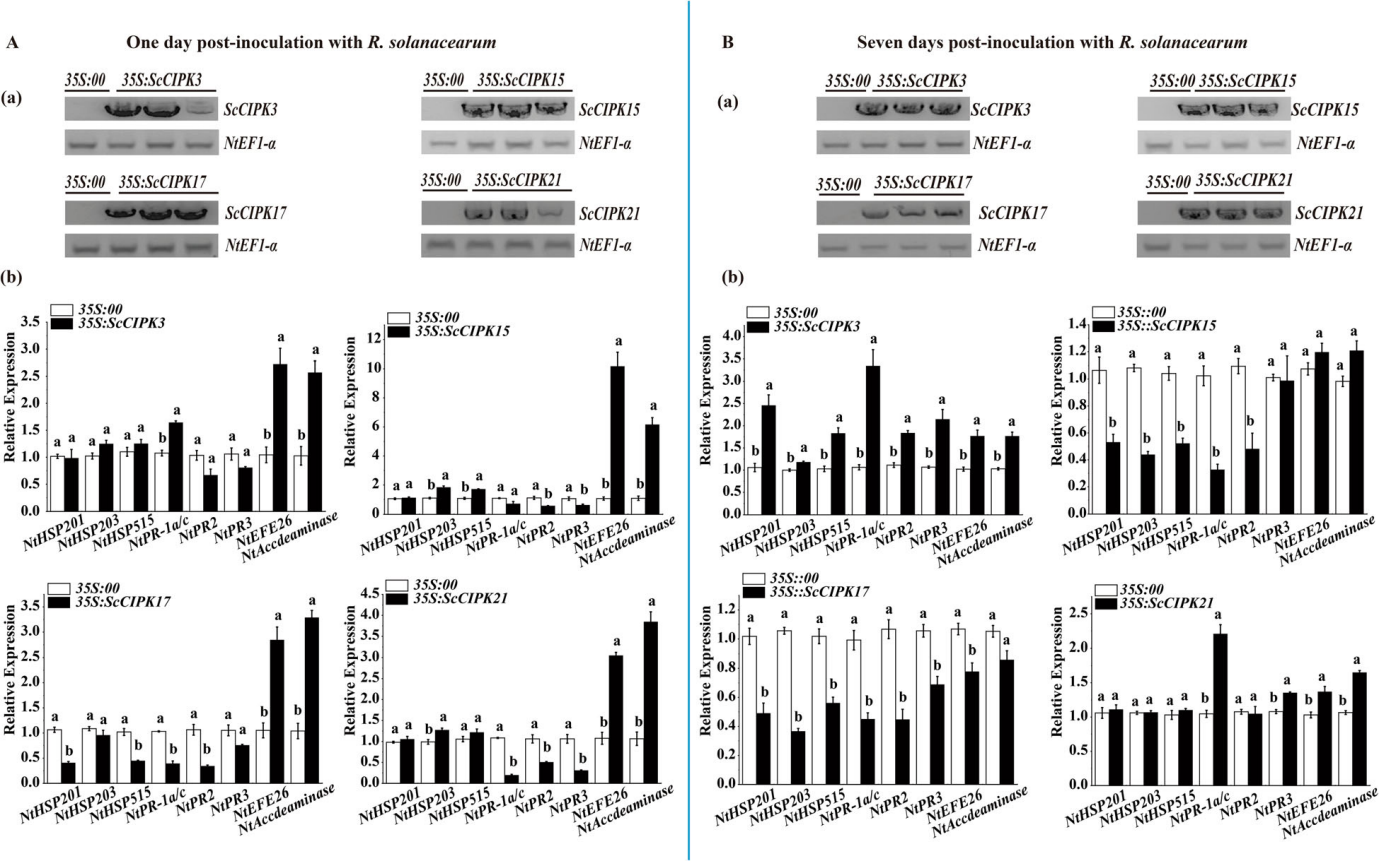
The effect of transient overexpression of *ScCIPKs* in *Nicotiana benthamiana* leaves after inoculation with *Ralstonia solanacearum*. A, RT-PCR analysis (**a**) (The gels were selected from the same gel with the same exposure, and the unmodified figure was shown in the Fig. S4-S7) and the relative transcript levels of the immunity-associated marker genes (**b**) in the *ScCIPKs* transiently expressed *N. benthamiana* challenged by *R. solanacearum* for 1 day. B, RT-PCR analysis (**a**) (The gels were selected from the same gel with the same exposure, and the unmodified figure was shown in the Fig. S4-S7) and the relative transcript levels of the immunity-associated marker genes (**b**) in the *ScCIPKs* transiently expressed *N. benthamiana* challenged by *R. solanacearum* for 7 days. *NtEF1-α* was used for normalization of the transcript levels. Mock, the *Agrobacterium* strain carrying *35S*::*00*. All of the data points were expressed as the mean ± SE (*n* = 3). Different lowercase letters indicate a significant difference between *35S*::*00* and *35S*::*ScCIPKs* in each gene, as determined by the Duncan’s new multiple range test (*p***-**value < 0.05)

After transient overexpression of *ScCIPKs* in *N. benthamiana* leaves, the expression levels of eight tobacco immunity associated marker genes were detected by qRT-PCR at 2 days post inoculation with *ScCIPKs* (Fig. 7), 1 day (Fig. 8A (b)), and 7 days (Fig. 8B (b)) post inoculation with *Ralstonia solanacearum* (Supplementary Fig. S4, Fig. S5, Fig. S6, and Fig. S7).

The transcript expression levels of ethylene synthesis dependent genes (*NtEFE26* and *NtAccdeaminase*) were upregulated in *ScCIPK15*, − *17* and − *21* except for *ScCIPK3* at 2 days post inoculation with *35S::ScCIPKs*. The expression of hypersensitive response (HR) marker genes (*NtHSR203* and *NtHSR515*) and SA-related gene *NtNPR2* was inhibited, whereas that of *NtHSR201*, *NtPR-1a/c*, and *NtNPR3* did not change in the control and *35S::ScCIPK3* leaves. In the *35S::ScCIPK15* leaves, the expression level of *NtNPR3* was upregulated, whereas that of the three HR maker genes did not change, and only *NtPR-1a/c* and *NtNPR2* were downregulated compared to the control. The transcript abundance of five genes (*NtHSR201*, *NtHSR203*, *NtHSR515*, *NtPR-1a/c*, and *NtNPR2*) decreased, and only *NtNPR3* did not change in the *35S::ScCIPK17* leaves. In the *35S::ScCIPK21* leaves, the expression of two HR marker genes (*NtHSR201* and *NtHSR515*) showed no significant difference compared to the control, whereas the other four genes (*NtHSR203*, *NtPR-1a/c*, *NtNPR2*, and *NtNPR3*) were downregulated.

One day after inoculation with *R. solanacearum* (Fig. 8A (b)), the ethylene synthesis dependent genes were also upregulated compared to the control in the four *35S::ScCIPKs* leaves. Except for the ethylene synthesis dependent genes, the expression of the *NtPR-1a/c* gene was only induced in the transient *ScCIPK3*-overexpressing leavess*. NtHSR515* was induced in the *35S::ScCIPK15* leaves. The HR marker gene, *NtHSR203,* was upregulated in both the *35S::ScCIPK15* and *35S::ScCIPK21* leaves.

After 7 days post infiltration with *R. solanacearum* (Fig. 8B (b)), seven genes (*NtHSR201*, *NtHSR203*, *NtHSR515*, *NtPR-1a/c*, *NtNPR2*, *NtNPR3*, and *NtEFE26*) were inhibited in *35S::ScCIPK17* leaves. Two SA-related genes (*NtPR-1a/c* and *NtNPR3*) and two ethylene synthesis dependent genes (*NtEFE26* and *NtAccdeaminase*) were upregulated in the *35S::ScCIPK21* leaves. All of the eight marker genes were upregulated in the *35S::ScCIPK1* leaves. However, in the *35S::ScCIPK15* leaves, three HR marker genes (*NtHSR201*, *NtHSR203*, and *NtHSR515*) and SA-related genes (*NtPR-1a/c* and *NtNPR2*) were downregulated.

## Discussion

The CIPK protein is a plant specific Ser/Thr protein kinase which can interact with calcium sensor CBL to form a complex signaling network mediating calcium signaling and various environmental stimuli [47, 48]. The *CIPK* gene family has been extensively studied in *A. thaliana* [11], as well as in other major crops, such as rice [49] and sorghum [50]. However, CIPK family genes in sugarcane have not been studies to date. Its significant function and lack of research into consideration, genome-wide and function analysis of CIPK family genes from sugarcane is imperative, and it may be used in the identification of resistance-related genes in sugarcane resistance breeding. This study, utilized the genome of *S. spontaneum*, and bioinformatics methods to determine the evolution of the *CIPK* gene family in *Saccharum*. Comprehensive molecular biology techniques were employed to verify the functional divergence of *CIPK* genes.

### Evolution of the *CIPK* gene family

In parallel to the results of phylogenetic analysis (Fig. 2), the gene structure of the *SsCIPKs* incurred substantial variations during evolution, with the number of introns varying from 0 to 15. *SsCIPK20* and *SsCIPK32* may have emerged after the first gene duplication event, with 12 introns for each. However, those genes that were inferred to have emerged after the sixth gene duplication event consisted of introns ranging in number from 0 to 3. Gene structures can evolve by the insertion and/or loss of introns [51]. In the present study, the evolution of gene structures in *SsCIPK* family genes involved intron losses. The results were consistent with the research on soybean *CIPK* family genes [43].

The composition of motifs can reflect the similarities and differences among gene family proteins. The proteins in the same clade of phylogenetic tree usually had similar motif compositions (Fig. 1). It is well know that in N-terminal of common CIPK proteins there was a conserved kinase domain with a putative activation loop between the DFG and APE residues [47, 52]. In the C-terminal, CIPK proteins usually had a unique regulatory domain with a conserved NAF/FISL motif [24, 26]. Some CIPKs even contained a protein phosphatase 2C binding domain designated as PPI [46, 53, 54]. In this study, five SsCIPKs did not have the motif 2 (DFG residues) and three SsCIPKs lost the motif 5 (APE residues), suggesting that the activation loop may lose in the SsCIPK proteins. Only SsCIPK11 did not have the motif 7 (NAF/FISL motif), which indicated that SsCIPK11 lost the NAF/FISL motif. Since the NAF/FISL motif was the key to regulating CBL-CIPK interaction [26], SsCIPK11 may not interact with CBLs. Here, nineteen SsCIPKs were found lacking motif 8 (PPI). The result implied that these SsCIPKs may not have the ability to interact with ABI2.

WGDs or polyploidy were considered as an important driving force in the evolution of organisms, especially for ancestral polyploidy in seed plants and angiosperms [55, 56]. In this study, 48 *CIPK* genes were identified in *S. spontaneum* genome. Figure 2 showed the phylogeny of the *SsCIPK* gene family, which may have undergone six gene duplication events from the LCA of *SsCIPK*. The two *SsCIPK* genes (*SsCIPK20* and − *32*) that were clustered in group I (subfamilies A and B) may have emerged after the first gene duplication, while 12 *SsCIPK* genes (*SsCIPK1*, −*2a*, −*2b*, − *5*, −*8a*, −*8b*, − *12*, − *14*, −*23a*, −*23b*, −*23c*, and − *24*) in group I (subfamilies C, D, and E) have appeared after the second duplication event. Besides, *SsCIPK17*, − *28*, and − *30* may have emerged after the third gene duplication event, and only *SsCIPK11* from the fourth, 18 genes (*SsCIPK4a*, −*4b*, −*4c*, −*4d*, −*4e*, −*7a*, −*7b*, − *9*, − *13*, − *16*, − *18*, − *19*, − *26*, −*27a*, −*27b*, −*29a*, −*29b*, and − *33*) from the fifth, while 12 *SsCIPKs* (*SsCIPK3,* − *6*, − *10*, − *15*, − *21*, −*22a*, −*22b*, −*22c*, −*25a*, −*25b*, −*31a* and − *31b*) from the sixth.

Except for WGDs, single-gene duplicates also play an important role in the formation of gene families [51]. Previous studies have shown that single-gene duplicates have higher levels of expression divergence, functional innovation, network rewiring and epigenetic changes than duplicated genes retained from WGDs [57–62]. On the basis of defined criteria, 16 pairs of duplicated genes were identified in this study. Five tandem duplication events occurred in chromosomes 2A, 2B, 4C and 7B. Meanwhile, a total 12 pairs of *SsCIPK* paralog genes were produced by segmental duplication events between chromosomes. These observations suggest that single-gene duplication events play important roles in the expansion of *SsCIPK* gene family in sugarcane. By estimating the divergence time of these 16 pairs of duplicated genes with an average duplication time of approximately 35.66 Mya, we hypothesize that these duplication events occurred after the divergence of the monocots and eudicots (165 Mya) [63]. Xi et al. [64] found that the tandem and segmental duplication events of grapevine *CIPK* genes might have happened around 17 Mya and 11 Mya, respectively. In addition, the divergence time of the *BrCIPK* paralogs was between 1.4457 and 28.1533 Mya, with an average duplication time of approximately 12.4194 Mya [65]. Our results implied that the gene duplication events of *CIPKs* in the monocots and dicots occurred after the divergence of monocots and eudicots. The Ka/Ks ratio of 15 out of 16 pairs was < 1, indicating that the duplicated genes were possibly under strong purifying selection pressure [66] and functional constraint in *S. spontaneum*. Strong purifying selection pressure also was reported in the *CIPKs* in turnip [65].

### Functional divergence of *ScCIPKs*

The *CIPK* genes play important roles in the responses to phytohormones and abiotic stress [22, 27, 32, 67]. To investigate the expression patterns of *ScCIPKs* under phytohormones stimulus and abiotic stress in sugarcane, qRT-PCR was used to analyze the expression of 10 *ScCIPK* genes under ABA, PEG and NaCl stress.

ABA is an important signaling molecule in biotic and abiotic stress responses [68]. In the present study, only *ScCIPK21* was upregulated at 3 h and 6 h under ABA stress, whereas the transcript levels of *ScCIPK1, − 2, − 3*, − *4*, *− 15*, *− 28,* and *-31* were downregulated. *ScCIPK2* and *ScCIPK20* did not show significant changes in expression at either time point tested. Previous studies have shown that *OsCIPK5* [49] and *TaCIPK31* [32] exhibited ABA-induced upregulation. In canola seedlings, *BnaCIPK6* was upregulated, *BnaCIPK3* was downregulated and the other 10 *BnaCIPKs* showed no significant changes under ABA [22].

Adequate evidence has demonstrated that *CIPK* genes play an important role in response to drought stress [12]. Several genes had been reported to be induced by drought stress, such as 4 *AtCIPKs* (*AtCIPK6*, − *9*, − *11*, and − *23*) [22] and 12 *OsCIPKs* (*OsCIPK1*, *− 2*, *− 5*, *− 6*, *− 14*, *− 17*, *− 19*, *− 23*, *− 24*, *− 25*, *− 31*, and *− 32*) [13]. *AtCIPK6* [69], *OsCIPK12* [49], and *OsCIPK23* [70] enhanced drought tolerance. Under PEG stress, the expression of 6 out of 10 *ScCIPKs* (*ScCIPK1*, *− 2, − 15*, *− 20*, − *21* and *− 28*) peaked at 24 h, whereas the other four (*ScCIPK3*, − *4*, *− 17*, and *− 31*) were downregulated. NaCl treatment upregulated *ScCIPK1, − 2*, and *− 28* expression, and downregulated *ScCIPK3*, *− 4*, *− 15*, − *20*, *− 21*, and *− 31* transcript expression. In eggplant (*Solanum melongena* L.), under NaCl stress, 11 *SmCIPKs* (*SmCIPK2, − 3, − 6, − 11, − 12, − 14, − 17, − 22, − 23, − 24*, and *− 25*) were upregulated, whereas transcript expression of *SmCIPK4, − 7*, and *− 20* was downregulated [71]. Sun et al. [32] found that under salt stress, *TaCIPK24* was upregulated, and overexpression of *TaCIPK24* improved salt tolerance in *Arabidopsis*. From what has been discussed above, the *ScCIPK* genes may have different roles in response to drought and salt stress.

Prokaryotic expression was performed to study the expression of four *ScCIPKs*. Previous studies have shown that overexpression of plant-stress-tolerant functional genes in *E. coli* cells enhances their growth under abiotic stresses [72–75]. In this study, the recombinant and control cells showed the similar growth on LB medium in overnight grown cultures. On 250 mM NaCl supplemented medium, except *ScCIPK21*, the recombinant cells showed increased number of colonies compared to control cells. However, no cell growth was observed on the 500 mM and 750 mM NaCl-supplemented medium. These results suggest that the recombinant proteins of *ScCIPK3, − 15*, and *− 17* only slightly enhance growth in NaCl stress condition, whereas *ScCIPK21* does not.

Transient overexpression is widely used to study the function of genes under biotic stresses [76, 77]. Figures 7 and 8 showed that defense-related genes are differentially expressed in *N. benthamiana* leaves that transiently express *ScCIPKs*. As for *ScCIPK3*, the defense-related genes were repressed or showed no significant changes after overexpression for 2 days (Fig. 7B), whereas *NtPR-1a/c*, *NtEF26*, and *NtAccdeminase* were upregulated at 1 day post inoculation with *R. solanacearum* (Fig. 8 A (b)). After 7 days post inoculation with *R. solanacearum*, seven out of eight immunity-associated marker genes were upregulated (Fig. 8 B (b)). The results suggest that *ScCIPK3* plays a role in the induction of immune-related genes during later stages. After 7 days post inoculation with *R. solanacearum*, the defense-related genes were downregulated or showed no significant changes in *ScCIPK15* and *ScCIPK17* expression (Fig. 8 B (b)), whereas two or three genes were upregulated at 2 days post inoculation with *ScCIPK17* or *ScCIPK15* (Fig. 7B) and at 1 day post inoculation with *R. solanacearum* (Fig. 8 A (b)), respectively. The ethylene synthesis-dependent genes (*NtEFE26* and *NtAccdeaminase*) were almost induced in the all of transiently overexpressed materials. Ethylene not only play as an internal signal regulator during plant growth and development, but also can respond to external adverse conditions (biotic and abiotic stresses) [78].

CIPKs are an important part of the Ca^2+^-mediated CBL-CIPK network and can specifically target CBLs to transduce the perceived calcium signal in response to diverse stimuli [21, 22]. Previous studies on the relationship between ethylene and CBL-CIPK networks have found that ethylene-responsive gene can be activated by the CBL-CIPK networks when the concentration of Ca^2+^ ions is increased [79]. Based on our results, we hypothesize that different *ScCIPK* genes work at various times of infection and may play a role in the response to external stressors through the ethylene synthesis pathway [79].

## Conclusions

In this study, 48 *SsCIPK* genes were identified from the *S. spontaneum* genome. These genes were uneven on the chromosomes. Base on the number of introns, these genes can be divided into intron-poor and intron-rich groups. The *SsCIPK* gene family not only underwent six gene duplication events, but also single-gene duplications. The Ka/Ks ratio showed that the duplicated genes of *SsCIPKs* were possibly under strong purifying selection pressure. Ten *ScCIPK* genes were cloned in sugarcane cultivar ROC22 (*Saccharum* spp. hybrid). Under ABA, PEG, and NaCl stress, 10 *CIPK* genes were differentially expressed in ROC22. A spot assay demonstrated that only *ScCIPK3*, − *15*, and − *17* could slightly enhance growth in NaCl stress condition, whereas *ScCIPK21* does not. The results of transient overexpression of *ScCIPKs* in *N. benthamiana* leaves suggested that the *ScCIPK* genes may respond against the attack of *R. solanacearum* through the ethylene synthesis pathway and function at different times of infection. These results provided information on the evolution and functional divergence of the *CIPK* gene family that may be utilized in breeding sugarcane cultivars with improved stress tolerance.

## Methods

### Plant materials and treatment

The sugarcane cultivar ROC22 (*Saccharum* spp. hybrid) was collected from the Key Laboratory of Sugarcane Biology and Genetic Breeding, Ministry of Agriculture (Fuzhou, China).

According to the method of Su et al. [80, 81]., uniform four-month-old tissue cultured plantlets of ROC22 were grown in water for 1 week and then treated with three exogenous treatments by root dipping at 28 °C with 16 h light and 8 h darkness, including 100 μM ABA, 25% PEG 8000, 250 mM NaCl. The whole plantlets treated by ABA were collected at 0 h, 3 h, 6 h and 12 h. The plantlets under 25% PEG and 250 mM NaCl treatments were collected at 0 h, 6 h, 12 h, and 24 h. Each treatment contained three biological replicates. The harvested samples were immediately frozen in liquid nitrogen and stored at − 80 °C until total RNA extraction.

### Identification of *CIPK* gene family members in *S. spontaneum* genome

The genome of *S. spontaneum* [42] was downloaded from the National Center for Biotechnology Information (NCBI, https://www.ncbi.nlm.nih.gov/) database. Hidden Markov Model (HMM) analysis was used for searching the *CIPK* genes. A total of 30 OsCIPKs [49] and 34 ZmCIPKs [27] protein sequences were aligned and used to construct a specific CIPK HMM using hmmbuild from the HMMER v3.2.1 suite. This new CIPK HMM was used, and all proteins with an E-value lower than 0.01 were selected. Then the HMM profile of NAF (PF03822), which was downloaded from Pfam protein database (http://pfam.sanger.ac.uk/) was used to further search the gene sets obtained from a previous screen. After removing all of the redundant sequences, the resulting putative CIPK protein sequences were submitted to CDD (https://www.ncbi.nlm.nih.gov/Structure/bwrpsb/bwrpsb.cgi), and Pfam protein database was applied to confirm the domain. Finally, the preserved sequences of *SsCIPKs* were named based on their locations on the chromosomes.

### Bioinformatics analysis

The *p*I and MW of each CIPK protein were predicted using ExPASy (http://web.expasy.org/protparam/). WOLF PSORT (https://www.genscript.com/wolf-psort.html) was used to predict the subcellular location of these CIPK proteins. The palmitoylation sites and myristoylation sites were predicted by CSS-Palm (http://csspalm.biocuckoo.org/). MapGene2Chrom (MG2C) software (http://mg2c.iask.in/mg2c_v2.1/) was applied to map the chromosomal positions of the SsCIPK genes. The exon-intron structures of *SsCIPK* genes were gathered from the GFF3 file of the *S. spontaneum* genome. Motifs prediction was performed using MEME (Multiple Em for Motif Elicitation) program (http://meme-suite.org/tools/meme). The optimized parameters were employed as follows: the number of repetitions, any; the maximum number of motifs, 20; and the optimal width of each motif, between 6 and 100 residues. The gene structures and motifs were merged using TBtools [82]. MEGA X (http://www.megasoftware.net/) was employed for phylogenetic analysis. Gene duplication was confirmed according to two criteria: (1) the length of the shorter aligned sequence covered > 70% of the longer sequence; and (2) the similarity of the two aligned sequences were > 70% [83, 84]. In the same chromosome, two genes separated by five or fewer genes within a < 100 kb chromosome fragment were identified as tandem duplicated genes [85]. All of the duplicated genes were mapped to chromosomes based on physical location information from the database of *S. spontaneum* genome using Circos [86]. KaKs_Calculator v2.0 was applied to calculate the Ka/Ks ratios using the maximum-likelihood MA method [87]. To assess the validity of the Ka and Ks that were calculated by this method, Fisher’s exact test for small samples was used [88]. The divergence time (T) was calculated by T = Ks/(2 × 6.1 × 10^− 9^) × 10^− 6^ Mya [66]. Clustal Omega (https://www.ebi.ac.uk/Tools/msa/clustalo/) was used to calculate the percent identity matrix between CIPK proteins in sugarcane.

### RNA extraction and first-strand cDNA synthesis

Refer to the manufacturer’s specifications, TRIzol® Reagent (Invitrogen, Carlsbad, CA, USA) was used to extract total RNA from all of the samples collected. A spectrophotometer (NanoVueplus, GE, USA) was used to measure absorbance at wavelengths of 260 nm and 280 nm, and RNA samples with an OD_260_/OD_280_ between 1.8 and 2.0 were selected for further analysis. DNase I (Promega, USA) was used to remove DNA contamination. For cloning, first-strand cDNA was synthesized using a RevertAid First Strand cDNA Synthesis Kit (Fermentas, Shanghai, China). For qRT-PCR analysis, the Prime-Script™ RT Reagent Kit (Perfect for Real Time) (TaKaRa, Dalian China) was used to synthesize the first-strand cDNA.

### Cloning of *ScCIPK* gene family and gateway entry vector construction

The CSA221G05 Maturing Sugarcane Stem Lambda ZIPLOX Library (MCS) *Saccharum* hybrid cultivar Q117 cDNA clone MCSA211C03 5′ similar to serine/threonine kinase, mRNA sequence (GenBank Accession No. CF577339.1) was used as a probe and the NCBI BlastN tool was used to retrieve homologous EST sequences in the sugarcane genome. The BioEdit Contig Assembly Program (CAP) was used to assemble one of the *ScCIPK* sequences (*ScCIPK15*). Three *ScCIPK* sequences (*ScCIPK1, ScCIPK17,* and *ScCIPK20*) were selected from our previous transcriptome data of sugarcane infected with sugarcane mosaic virus [89]. The other six *ScCIPK* sequences (*ScCIPK2, ScCIPK3, ScCIPK4, ScCIPK21, ScCIPK28,* and *ScCIPK31*) were screened from our previous transcriptome data of sugarcane infected with smut fungus [90]. Primer 5.0 and the NCBI primer designing tool (http://www.ncbi.nlm.nih.gov/tools/primer-blast/) were employed to design the specific primers for cloning the target genes (Supplementary Table S5). The system of RT-PCR reactions was constructed by the specifications for *Ex* Taq (TaKaRa, Dalian, China) and *LA* Taq (TaKaRa, Dalian, China). The amplification reactions are shown in Supplementary Table S6. Then, 1% agarose gel electrophoresis was performed to detect the PCR products. The right PCR products were selected, purified, ligated into the pMD-19-T vector, transformed into *E. coli* DH5α and sequenced (Sangon, Shanghai, China).

The primers of the Gateway entry vector were used to amplify the *ScCIPK* ORFs from pMD19-T-*ScCIPKs* with Gateway entry adapters attB1 and attB2 (Supplementary Table S5**)**. According to the manufacturer’s instructions, Gateway® BP Clonase™ II Enzyme Mix (Invitrogen) was used to ligate the PCR amplification products, which were gel**-**purified into the Gateway^@^ donor vector pDONR221 (Invitrogen). Then, the BP reaction mixtures were transformed into *E. coli* DH5α cells and sequenced (Sangon, Shanghai, China). The verified pDONR221-*ScCIPK* plasmids were chosen and used in the construction of expression vectors.

### Expression pattern of *ScCIPKs* under ABA, PEG, and NaCl stress

Using the 7500 qRT-PCR system (Applied Biosystems, South San Francisco, CA, USA), the relative expression levels of *ScCIPKs* under different exogenous stresses were assessed. The qRT-PCR primers of *ScCIPKs* were designed using Beacon Designer 8.12 software. The Cullin (*CUL*) [91] and Clathrin adaptor complex (*CAC*) [91] genes were used to normalize relative transcript levels. The qRT-PCR reaction system was prepared using the SYBR Green Master Mix (TaKaRa), following the manufacturer’s instructions. Each qRT-PCR was repeated thrice, and the reaction conditions were listed as follows: 50 °C for 2 min, 95 °C for 10 min, followed by 40 cycles of 95 °C for 15 s and 60 °C for 1 min. The qRT-PCR data was analyzed using the 2^-ΔΔCt^method [92]. Statistical analysis was conducted using Data Processing System v9.50 software (China). Significance (*p* < 0.05) was calculated using one-way ANOVA, followed by Duncan’s new multiple range test. All of the primers used in qRT-PCR are listed in Supplementary Table S5.

### Salinity stress tolerance assay using transformed *E. coli* BL21 (DE3) cells

The prokaryotic expressive vectors of pEZYHb-*ScCIPKs* were constructed using LR. The recombinant plasmids of pEZYHb-*ScCIPKs* were transformed into *E. coli* BL21 (DE3) competent cells, and the empty vector pEZYHb was transformed into *E. coli* BL21 and served as control. For the salt tolerance assay, an additional 250 mM, 500 mM, and 750 mM concentration gradient of NaCl was added to the LB media. When the transformed *E. coli* BL21 cells were grown to an OD_600_ of 0.6, 1.0 mmol L^− 1^ isopropyl β-D-thiogalactoside (IPTG) was added to induce protein production and further grown for 12 h at 37 °C. Cell density was adjusted to an OD_600_ = 0.6, and then samples were diluted to 10^− 3^- and 10^− 4^-fold using LB medium [93]. Ten microliters of each of the 10^− 3^-and 10^− 4^-fold dilutions of the sample were spotted onto the LB agar plates.

### The role of the *ScCIPK* genes in response to *Ralstonia solanacearum* infection

The experiment involved two groups, one overexpressed the target gene for 2 days, and the other overexpressed target gene for 1 day then inoculated with tobacco bacteria *Ralstonia solanacearum* for 1 day and 7 days. Overexpression vectors pEarleyGate 203-*ScCIPKs* were constructed using the Gateway cloning technique, then transformed into *Agrobacterium* strain GV1301 competent cells. GV1301 cells with empty vector pEarleyGate 203 served as control. After incubation, the cells that contained pEarleyGate 203-*ScCIPKs* and pEarleyGate 203 were centrifuged and resuspended in induction medium (10 mM MES, 10 mM MgCl_2_, 200 μM acetosyringone, pH 5.6) at an OD_600_ of 0.8 [94]. *R. solanacearum* were cultured overnight in potato dextrose water (PDW) liquid medium at 200 rpm and 28 °C, then resuspended in 10 mM magnesium chloride (MgCl_2_) solution and injected into the one-day overexpression *N. benthamiana* leaves. All of the injected leaves were collected for RNA extraction to analyze the expression level of *ScCIPKs* in *N. benthamiana* semi-quantitatively using with the specific primers (Supplementary Table S5). The transcript levels of eight tobacco immunity-associated marker genes [HR marker genes *NtHSR201*, *NtHSR203* and *NtHSR515*, SA-related gene *NtPR-1a/c*, *NtPR2*, and *NtPR3*, ET synthesis-dependent genes *NtEFE26* and *NtAccdeaminase*, Supplementary Table S5] were detected by qRT-PCR. The 2^-ΔΔCt^method [92] and DPS v9.50 software (China) were used to analyzed the qRT-PCR data. One-way ANOVA, followed by Duncan’s new multiple range test was used to calculate data significance (*p* < 0.05). All of the primers used in qRT-PCR are listed in Supplementary Table S5.

## Supplementary Information

**Additional file 1: Figure S1.** Chromosomal distribution of *SsCIPKs*. The scale bar on the left indicated the length (Mb) of sugarcane chromosomes. **Figure S2.** Analysis and distribution of conserved motifs in SsCIPK proteins. **Figure S3.** Phylogenetic analysis of CIPK proteins from *S. spontaneum* and *Saccharum* spp*.* hybrid (ROC22). Red triangles represent ScCIPK proteins. **Figure S4.** RT-PCR analysis of *ScCIPK3* in the *N. benthamiana* leaves 2 days after infiltration with *Agrobacterium* strain GV3101 that carried the vector *35S::00* or *35S::ScCIPK3*, in the *ScCIPK3* transiently expressed *N. benthamiana* challenged by *R. solanacearum* for 1 day, and in the *ScCIPK3* transiently expressed *N. benthamiana* challenged by *R. solanacearum* for 7 days. **Figure S5.** RT-PCR analysis of *ScCIPK15* in the *N. benthamiana* leaves 2 days after infiltration with *Agrobacterium* strain GV3101 that carried the vector *35S::00* or *35S::ScCIPK15*, in the *ScCIPK15* transiently expressed *N. benthamiana* challenged by *R. solanacearum* for 1 day, and in the *ScCIPK15* transiently expressed *N. benthamiana* challenged by *R. solanacearum* for 7 days. **Figure S6.** RT-PCR analysis of *ScCIPK17* in the *N. benthamiana* leaves 2 days after infiltration with *Agrobacterium* strain GV3101 that carried the vector *35S::00* or *35S::ScCIPK17*, in *the ScCIPK17* transiently expressed *N. benthamiana* challenged by *R. solanacearum* for 1 day, and in *the ScCIPK17* transiently expressed *N. benthamiana* challenged by *R. solanacearum* for 7 days. **Figure S7.** RT-PCR analysis of *ScCIPK21* in the *N. benthamiana* leaves 2 days after infiltration with *Agrobacterium* strain GV3101 that carried the vector *35S::00* or *35S::ScCIPKs*, in the *ScCIPK21* transiently expressed *N. benthamiana* challenged by *R. solanacearum* for 1 day, and in the *ScCIPK21* transiently expressed *N. benthamiana* challenged by *R. solanacearum* for 7 days.

**Additional file 2: Table S1.** The information of CIPK sequences in *S. spontaneum*.

**Additional file 3: Table S2.** The prediction of Palmitoylation and Myristoylation sites.

**Additional file 4: Table S3.** List of identified *CIPK* genes in other plant species.

**Additional file 5: Table S4.** Percentage of identity between CIPK proteins in sugarcane was calculated using Clustal Omega.

**Additional file 6: Table S5.** Primers used in this study.

**Additional file 7: Table S6.** The amplification reaction procedures for cloning of sugarcane *ScCIPKs*.

## Data Availability

The sequences of ten *CIPK* genes from sugarcane analysed during the current study are available in the NCBI repository with the Accession Numbers of KX013381, KX013387, KX013382, KX013378, KX013384, KX013386, KX013380, KX013379, KX013385 and KX013383. The data of *Saccharum spontaneum* genome can be downloaded from the following link: http://www.life.illinois.edu/ming/downloads/Spontaneum_genome/. All the other data supporting the conclusions of this article are within the paper.

## Abbreviations

CIPK: CBL-interacting protein kinases
CBL: Calcineurin B-like
LCA: Last common ancestor
WGD: Whole-genome duplication
ABA: Abscisic acid
PEG: Polyethylene glycol
HMM: Hidden markov model
NaCl: Sodium chloride
aa: Amino acid
MW: Molecular weight
pI: Isoelectric points
ML: Maximum likelihood
Ka: Nonsynonymous
Ks: Synonymous
PPI: Protein-phosphatase interaction
ABI2: Abscisic acid-insensitive 2
*CUL*: Cullin
*CAC*: Clathrin adaptor complex
MG2C: MapGene2Chrom
IPTG: Isopropyl β-D-thiogalactoside
PDW: Potato dextrose water
MgCl_2_: Magnesium chloride
HR: Hypersensitive response
CAP: Contig assembly program
RT-PCR: Reverse transcription-polymerase chain reaction
qRT-PCR: Quantitative reverse transcription polymerase chain reaction
SE: Standard error

## References

[1] Bohnert HJ, Gong Q, Li P, Ma S (2006). Unraveling abiotic stress tolerance mechanisms--getting genomics going. Curr Opin Plant Biol.

[2] Fujita M, Fujita Y, Noutoshi Y, Takahashi F, Narusaka Y, Yamaguchi-Shinozaki K, Shinozaki K (2006). Crosstalk between abiotic and biotic stress responses: a current view from the points of convergence in the stress signaling networks. Curr Opin Plant Biol.

[3] Albrecht V, Weinl S, Blazevic D, D'Angelo C, Batistic O, Kolukisaoglu U, Bock R, Schulz B, Harter K, Kudla J. The calcium sensor CBL1 integrates plant responses to abiotic stresses. Plant J. 2003;36(4):457–70. 10.1046/j.1365-313x.2003.01892.x 14617077

[4] Jayasekaran K, Kim K-N, Vivekanandan M, Shin JS, Ok SH (2006). Novel calcium-binding GTPase (AtCBG) involved in ABA-mediated salt stress signaling in Arabidopsis. Plant Cell Rep.

[5] Xiong L, Yang Y (2003). Disease resistance and abiotic stress tolerance in rice are inversely modulated by an abscisic acid-inducible mitogen-activated protein kinase. Plant Cell.

[6] Hansen JD, Pyee J, Xia Y, Wen TJ, Robertson DS, Kolattukudy PE, Nikolau BJ, Schnable PS (1997). The *glossy1* locus of maize and an epidermis-specific cDNA from *Kleinia odora* define a class of receptor-like proteins required for the normal accumulation of cuticular waxes. Plant Physiol.

[7] Xia Y, Nikolau BJ, Schnable PS (1997). Developmental and hormonal regulation of the *Arabidopsis CER2* gene that codes for a nuclear-localized protein required for the normal accumulation of cuticular waxes. Plant Physiol.

[8] Xu X, Dietrich CR, Delledonne M, Xia Y, Wen TJ, Robertson DS, Nikolau BJ, Schnable PS (1997). Sequence analysis of the cloned *glossy8* gene of maize suggests that it may code for a beta-ketoacyl reductase required for the biosynthesis of cuticular waxes. Plant Physiol.

[9] Tanaka H, Onouchi H, Kondo M, Haranishimura I, Nishimura M, Machida C, Machida Y (2001). A subtilisin-like serine protease is required for epidermal surface formation in *Arabidopsis* embryos and juvenile plants. Development.

[10] Chen X, Goodwin SM, Boroff VL, Liu X, Jenks MA (2003). Cloning and characterization of the *WAX2* gene of *Arabidopsis* involved in cuticle membrane and WAX production. Plant Cell.

[11] Kolukisaoglu U, Weinl S, Blazevic D, Batistic O, Kudla J (2004). Calcium sensors and their interacting protein kinases: genomics of the Arabidopsis and rice CBL-CIPK signaling networks. Plant Physiol.

[12] Hu W, Xia Z, Yan Y, Ding Z, Tie W, Wang L, Zou M, Wei Y, Lu C, Hou X (2015). Genome-wide gene phylogeny of CIPK family in cassava and expression analysis of partial drought-induced genes. Front Plant Sci.

[13] Kanwar P, Sanyal SK, Tokas I, Yadav AK, Pandey A, Kapoor S, Pandey GK (2014). Comprehensive structural, interaction and expression analysis of CBL and CIPK complement during abiotic stresses and development in rice. Cell Calcium.

[14] Evans NH, Mcainsh MR, Hetherington AM (2001). Calcium oscillations in higher plants. Curr Opin Plant Biol.

[15] Harper JF (2001). Dissecting calcium oscillators in plant cells. Trends Plant Sci.

[16] Knight H, Knight MR (2001). Abiotic stress signalling pathways: specificity and cross-talk. Trends Plant Sci.

[17] Chung E, Park JM, Oh S, Joung YH, Lee S, Choi D (2004). Molecular and biochemical characterization of the *Capsicum annuum calcium-dependent protein kinase 3* (*CaCDPK3*) gene induced by abiotic and biotic stresses. Planta.

[18] Snedden WA, Fromm H (1998). Calmodulin, calmodulin-related proteins and plant responses to the environment. Trends Plant Sci.

[19] Luan S, Kudla J, Rodriguez-Concepcion M, Yalovsky S, Gruissem W (2002). Calmodulins and calcineurin B-like proteins: calcium sensors for specific signal response coupling in plants. Plant Cell.

[20] Sanders D, Pelloux J, Brownlee C, Harper JF (2002). Calcium at the crossroads of signaling. Plant Cell.

[21] Deng X, Hu W, Wei S, Zhou S, Zhang F, Han J, Chen L, Li Y, Feng J, Fang B (2013). *TaCIPK29*, a CBL-interacting protein kinase gene from wheat, confers salt stress tolerance in transgenic tobacco. PLoS One.

[22] Zhang H, Yang B, Liu WZ, Li H, Wang L, Wang B, Deng M, Liang W, Deyholos MK, Jiang YQ (2014). Identification and characterization of CBL and CIPK gene families in canola (*Brassica napus* L.). BMC Plant Biol.

[23] Gribskov M, Harper JF, Choi JH, Kudla J, Luan S, Nimmo HG, Sussman MR, Zhu JK, Harmon AC (2003). The *Arabidopsis* CDPK-SnRK superfamily of protein kinases. Plant Physiol.

[24] Guo Y, Halfter U, Ishitani M, Zhu JK (2001). Molecular characterization of functional domains in the protein kinase SOS2 that is required for plant salt tolerance. Plant Cell.

[25] Gong D, Guo Y, Jagendorf AT, Zhu JK (2002). Biochemical characterization of the *Arabidopsis* protein kinase SOS2 that functions in salt tolerance. Plant Physiol.

[26] Albrecht V, Ritz O, Linder S, Harter K, Kudla J (2001). The NAF domain defines a novel protein–protein interaction module conserved in Ca^2+^-regulated kinases. EMBO J.

[27] Chen X, Gu Z, Xin D, Liang H, Liu C, Ji H, Ma B, Zhang H (2011). Identification and characterization of putative *CIPK* genes in maize. J Genet Genomics.

[28] Luo Q, Wei Q, Wang R, Zhang Y, Zhang F, He Y, Yang G, He G (2018). Ectopic expression of *BdCIPK31* confers enhanced low-temperature tolerance in transgenic tobacco plants. Acta Biochim Biophys Sin.

[29] Luo Q, Wei Q, Wang R, Zhang Y, Zhang F, He Y, Zhou S, Feng J, Yang G, He G (2017). BdCIPK31, a calcineurin B-like protein-interacting protein kinase, regulates plant response to drought and salt stress. Front Plant Sci.

[30] Zhao J, Sun Z, Zheng J, Guo X, Dong Z, Huai J, Gou M, He J, Jin Y, Wang J (2009). Cloning and characterization of a novel CBL-interacting protein kinase from maize. Plant Mol Biol.

[31] Shilpi M, Pandey GK, Narendra T (2008). Calcium- and salt-stress signaling in plants: shedding light on SOS pathway. Arch Biochem Biophys.

[32] Sun T, Wang Y, Wang M, Li T, Zhou Y, Wang X, Wei S, He G, Yang G (2015). Identification and comprehensive analyses of the CBL and CIPK gene families in wheat ( *Triticum aestivum* L.). BMC Plant Biol.

[33] Quan-Sheng Q, Yan G, Dietrich MA, Schumaker KS, Jian-Kang Z (2002). Regulation of SOS1, a plasma membrane Na^+^/H^+^ exchanger in *Arabidopsis thaliana*, by SOS2 and SOS3. Proc Natl Acad Sci U S A.

[34] Xu J, Li H-D, Chen L-Q, Wang Y, Liu L-L, He L, Wu W-H (2006). A protein kinase, interacting with two calcineurin B-like proteins, regulates K^+^ transporter AKT1 in *Arabidopsis*. Cell.

[35] Li J, Long Y, Qi G-N, Li J, Xu Z-J, Wu W-H, Wang Y (2014). The Os-AKT1 channel is critical for K^+^ uptake in rice roots and is modulated by the rice CBL1-CIPK23 complex. Plant Cell.

[36] Tripathi V, Parasuraman B, Laxmi A, Chattopadhyay D (2009). CIPK6, a CBL-interacting protein kinase is required for development and salt tolerance in plants. Plant J.

[37] Priji PJ, Hemaprabha G (2015). Sugarcane specific drought responsive candidate genes belonging to ABA dependent pathway identified from basic species clones of *Saccharum* sp. and *Erianthus* sp. Sugar Tech.

[38] Farani TF, Gentile A, Tavares RG, Ribeiro C, Menossi M (2015). Characterization of a protein-protein interaction network of the CBL-interacting protein kinase 8 from sugarcane. Gen Mol Res.

[39] Zhou D, Liu X, Gao S, Guo J, Su Y, Ling H, Wang C, Li Z, Xu L, Que Y (2018). Foreign *cry1Ac* gene integration and endogenous borer stress-related genes synergistically improve insect resistance in sugarcane. BMC Plant Biol.

[40] Suprasanna P, Patade VY, Desai NS, Devarumath RM, Kawar PG, Pagariya MC, Ganapathi A, Manickavasagam M, Babu KH (2011). Biotechnological developments in sugarcane improvement: an overview. Sugar Tech.

[41] Hoarau JY, Grivet L, Offmann B, Raboin LM, Diorflar JP, Payet J, Hellmann M, D'Hont A, Glaszmann JC (2002). Genetic dissection of a modern sugarcane cultivar (*Saccharum* spp.).II. Detection of QTLs for yield components. Theor Appl Genet.

[42] Zhang J, Zhang X, Tang H, Zhang Q, Hua X, Ma X, Zhu F, Jones T, Zhu X, Bowers J (2018). Allele-defined genome of the autopolyploid sugarcane *Saccharum spontaneum* L. Nat Genet.

[43] Zhu K, Chen F, Liu J, Chen X, Hewezi T, Cheng ZM (2016). Evolution of an intron-poor cluster of the CIPK gene family and expression in response to drought stress in soybean. Sci Rep.

[44] Weinl S, Kudla J (2009). The CBL-CIPK Ca^2+^-decoding signaling network: function and perspectives. New Phytol.

[45] Yu Y, Xia X, Yin W, Zhang H (2007). Comparative genomic analysis of CIPK gene family in *Arabidopsis* and *Populus*. Plant Growth Regul.

[46] Ohta M, Guo Y, Halfter U, Zhu JK (2003). A novel domain in the protein kinase SOS2 mediates interaction with the protein phosphatase 2C ABI2. Proc Natl Acad Sci U S A.

[47] Batistic O, Kudla J (2004). Integration and channeling of calcium signaling through the CBL calcium sensor/CIPK protein kinase network. Planta.

[48] Pandey GK (2008). Emergence of a novel calcium signaling pathway in plants: CBL-CIPK signaling network. Physiol Mol Biol Plants.

[49] Xiang Y, Huang Y, Xiong L (2007). Characterization of stress-responsive *CIPK* genes in rice for stress tolerance improvement. Plant Physiol.

[50] Li LB, Zhang YR, Liu KC, Ni ZF, Fang ZJ, Sun QX, Gao JW (2010). Identification and bioinformatics analysis of *SnRK2* and *CIPK* family genes in Sorghum. Agric Sci Chin.

[51] Hu W, Hua X, Zhang Q, Wang J, Shen Q, Zhang X, Wang K, Yu Q, Lin YR, Ming R (2018). New insights into the evolution and functional divergence of the SWEET family in *Saccharum* based on comparative genomics. BMC Plant Biol.

[52] Chaves-Sanjuan A, Sanchez-Barrena MJ, Gonzalez-Rubio JM, Moreno M, Ragel P, Jimenez M, Pardo JM, Martinez-Ripoll M, Quintero FJ, Albert A (2014). Structural basis of the regulatory mechanism of the plant CIPK family of protein kinases controlling ion homeostasis and abiotic stress. Proc Natl Acad Sci U S A.

[53] Lee SC, Lan WZ, Kim BG, Li L, Cheong YH, Pandey GK, Lu G, Buchanan BB, Luan S (2007). A protein phosphorylation/dephosphorylation network regulates a plant potassium channel. Proc Natl Acad Sci U S A.

[54] Sánchez-Barrena MJ, Fujii H, Angulo I, Martínez-Ripoll M, Zhu JK, Albert A (2007). The structure of the C-terminal domain of the protein kinase AtSOS2 bound to the calcium sensor AtSOS3. Mol Cell.

[55] Jiao Y, Wickett NJ, Ayyampalayam S, Chanderbali AS, Landherr L, Ralph PE, Tomsho LP, Hu Y, Liang H, Soltis PS (2011). Ancestral polyploidy in seed plants and angiosperms. Nature.

[56] Jiao Y, Li J, Tang H, Paterson AH (2014). Integrated syntenic and phylogenomic analyses reveal an ancient genome duplication in monocots. Plant Cell.

[57] Wang Y, Wang X, Tang H, Tan X, Ficklin SP, Feltus FA, Paterson AH (2011). Modes of gene duplication contribute differently to genetic novelty and redundancy, but show parallels across divergent angiosperms. PLoS One.

[58] Wang Y, Wang X, Lee TH, Mansoor S, Paterson AH (2013). Gene body methylation shows distinct patterns associated with different gene origins and duplication modes and has a heterogeneous relationship with gene expression in *Oryza sativa* (rice). New Phytol.

[59] Li Z, Zhang H, Ge S, Gu X, Gao G, Luo J (2009). Expression pattern divergence of duplicated genes in rice. BMC Bioinformatics.

[60] Casneuf T, De Bodt S, Raes J, Maere S, Van de Peer Y (2006). Nonrandom divergence of gene expression following gene and genome duplications in the flowering plant *Arabidopsis thaliana*. Genome Biol.

[61] Hakes L, Pinney JW, Lovell SC, Oliver SG, Robertson DL (2007). All duplicates are not equal: the difference between small-scale and genome duplication. Genome Biol.

[62] Guan Y, Dunham MJ, Troyanskaya OG (2007). Functional analysis of gene duplications in *Saccharomyces cerevisiae*. Genetics.

[63] Cheng J, Khan MA, Qiu WM, Li J, Zhou H, Zhang Q, Guo W, Zhu T, Peng J, Sun F (2012). Diversification of genes encoding granule-bound starch synthase in monocots and dicots is marked by multiple genome-wide duplication events. PLoS One.

[64] Xi Y, Liu J, Dong C, Cheng ZM (2017). The CBL and CIPK gene family in grapevine (*Vitis vinifera*): genome-wide analysis and expression profiles in response to various abiotic stresses. Front Plant Sci.

[65] Yin X, Wang Q, Chen Q, Xiang N, Yang Y, Yang Y (2017). Genome-wide identification and functional analysis of the calcineurin B-like protein and calcineurin B-like protein-interacting protein kinase gene families in Turnip (*Brassica rapa var. rapa*). Front Plant Sci.

[66] Lynch M, Conery JS (2000). The evolutionary fate and consequences of duplicate genes. Science.

[67] Yu Q, An L, Li W (2014). The CBL-CIPK network mediates different signaling pathways in plants. Plant Cell Rep.

[68] Xiong LM, Schumaker KS, Zhu JK (2002). Cell signaling during cold, drought, and salt stress. Plant Cell.

[69] Tsou PL, Sang YL, Allen NS, Winter-Sederoff H, Robertson D (2012). An ER-targeted calcium-binding peptide confers salt and drought tolerance mediated by CIPK6 in *Arabidopsis*. Planta.

[70] Omo-Ikerodah E (2008). Calcineurin B-like interacting protein kinase OsCIPK23 functions in pollination and drought stress responses in rice (*Oryza sativa* L.). J Genet Genomics.

[71] Li J, Jiang MM, Ren L, Liu Y, Chen HY (2016). Identification and characterization of *CBL* and *CIPK* gene families in eggplant (*Solanum melongena* L.). Mol Gen Genomics.

[72] Chaurasia N, Mishra Y, Rai LC (2008). Cloning expression and analysis of phytochelatin synthase (*pcs*) gene from *Anabaena* sp. PCC 7120 offering multiple stress tolerance in *Escherichia coli*. Biochem Biophys Res Commun.

[73] Guo XH, Jiang J, Wang BC, Li HY, Wang YC, Yang CP, Liu GF (2010). ThPOD3, a truncated polypeptide from *Tamarix hispida*, conferred drought tolerance in *Escherichia coli*. Mol Biol Rep.

[74] Liu Y, Zheng Y (2005). PM2, a group 3 LEA protein from soybean, and its 22-mer repeating region confer salt tolerance in *Escherichia coli*. Biochem Biophys Res Commun.

[75] Gupta K, Agarwal PK, Reddy MK, Jha B (2010). SbDREB2A, an a-2 type DREB transcription factor from extreme halophyte *Salicornia brachiata* confers abiotic stress tolerance in *Escherichia coli*. Plant Cell Rep.

[76] Li Y, Li Q, Guo G, He T, Gao R, Faheem M, Huang J, Lu R, Liu C (2018). Transient overexpression of *HvSERK2* improves barley resistance to powdery mildew. Int J Mol Sci.

[77] Liu F, Huang N, Wang L, Ling H, Sun T, Ahmad W, Muhammad K, Guo J, Xu L, Gao S (2017). A novel L-ascorbate peroxidase 6 gene, *ScAPX6*, plays an important role in the regulation of response to biotic and abiotic stresses in sugarcane. Front Plant Sci.

[78] Wang KL-C, Li H, Ecker JR (2002). Ethylene biosynthesis and signaling networks. Plant Cell.

[79] Blokhina O, Fagerstedt KV, Mancuso S, Shabala S (2010). Oxygen Deprivation, Metabolic Adaptations and Oxidative Stress. Waterlogging Signalling and Tolerance in Plants.

[80] Su W, Huang L, Ling H, Mao H, Huang N, Su Y, Ren Y, Wang D, Xu L, Muhammad K (2020). Sugarcane calcineurin B-like (CBL) genes play important but versatile roles in regulation of responses to biotic and abiotic stresses. Sci Rep.

[81] Su W, Ren Y, Wang D, Su Y, Feng J, Zhang C, Tang H, Xu L, Muhammad K, Que Y (2020). The alcohol dehydrogenase gene family in sugarcane and its involvement in cold stress regulation. BMC Genomics.

[82] Chen C, Chen H, Zhang Y, Thomas HR, Frank MH, He Y, Xia R (2020). TBtools: An integrative toolkit developed for interactive analyses of big biological data. Mol Plant.

[83] Gu Z, Cavalcanti A, Chen FC, Bouman P, Li WH (2002). Extent of gene duplication in the genomes of drosophila, nematode, and yeast. Mol Biol Evol.

[84] Yang S, Zhang X, Yue JX, Tian D, Chen JQ (2008). Recent duplications dominate NBS-encoding gene expansion in two woody species. Mol Gen Genomics.

[85] Wang L, Guo K, Li Y, Tu Y, Hu H, Wang B, Cui X, Peng L (2010). Expression profiling and integrative analysis of the CESA/CSL superfamily in rice. BMC Plant Biol.

[86] Krzywinski M, Schein J, Birol I, Connors J, Gascoyne R, Horsman D, Jones SJ, Marra MA (2009). Circos: an information aesthetic for comparative genomics. Genome Res.

[87] Posada D (2003). Using MODELTEST and PAUP* to select a model of nucleotide substitution. Current protocols in bioinformatics.

[88] Wang DP, Wan HL, Zhang S, Yu J (2009). Gamma-MYN: a new algorithm for estimating Ka and Ks with consideration of variable substitution rates. Biol Direct.

[89] Ling H, Huang N, Wu Q, Su Y, Peng Q, Ahmed W, Gao S, Su W, Que Y, Xu L (2018). Transcriptional insights into the sugarcane-*sorghum mosaic virus* interaction. Trop Plant Biol.

[90] Que Y, Su Y, Guo J, Wu Q, Xu L (2014). A global view of transcriptome dynamics during *Sporisorium scitamineum* challenge in sugarcane by RNA-Seq. PLoS One.

[91] Ling H, Wu QB, Guo JL, Xu LP, Que YX (2014). Comprehensive selection of reference genes for gene expression normalization in sugarcane by real time quantitative RT-PCR. PLoS One.

[92] Livak KJ, Schmittgen TD (2001). Analysis of relative gene expression data using real-time quantitative PCR and the 2^−ΔΔCT^ method. Methods.

[93] Guo JL, Xu LP, Fang JP, Su YC, Fu HY, Que YX, Xu JS (2012). A novel dirigent protein gene with highly stem-specific expression from sugarcane, response to drought, salt and oxidative stresses. Plant Cell Rep.

[94] Ifnan Khan M, Zhang Y, Liu Z, Hu J, Liu C, Yang S, Hussain A, Furqan Ashraf M, Noman A, Shen L (2018). *CaWRKY40b* in pepper acts as a negative regulator in response to *Ralstonia solanacearum* by directly modulating defense genes including *CaWRKY40*. Int J Mol Sci.

