# Supplementary material for: New insights into the evolution and functional divergence of the CIPK gene family in Saccharum
Source: BMC Genomics. 2020 Dec 7;21:868. doi: 10.1186/s12864-020-07264-9 (PMC7720545; doi:10.1186/s12864-020-07264-9)
Supplement: Supplementary file 1 — Additional file 1: Figure S1. Chromosomal distribution of SsCIPKs. The scale bar on the left indicated the length (Mb) of sugarcane chromosomes. Figure S2. Analysis and distribution of conserved motifs in SsCIPK proteins. Figure S3. Phylogenetic analysis of CIPK proteins from S. spontaneum and Saccharum spp. hybrid (ROC22). Red triangles represent ScCIPK proteins. Figure S4. RT-PCR analysis of ScCIPK3 in the N. benthamiana leaves 2 days after infiltration with Agrobacterium strain GV3101 that carried the vector 35S::00 or 35S::ScCIPK3, in the ScCIPK3 transiently expressed N. benthamiana challenged by R. solanacearum for 1 day, and in the ScCIPK3 transiently expressed N. benthamiana challenged by R. solanacearum for 7 days. Figure S5. RT-PCR analysis of ScCIPK15 in the N. benthamiana leaves 2 days after infiltration with Agrobacterium strain GV3101 that carried the vector 35S::00 or 35S::ScCIPK15, in the ScCIPK15 transiently expressed N. benthamiana challenged by R. solanacearum for 1 day, and in the ScCIPK15 transiently expressed N. benthamiana challenged by R. solanacearum for 7 days. Figure S6. RT-PCR analysis of ScCIPK17 in the N. benthamiana leaves 2 days after infiltration with Agrobacterium strain GV3101 that carried the vector 35S::00 or 35S::ScCIPK17, in the ScCIPK17 transiently expressed N. benthamiana challenged by R. solanacearum for 1 day, and in the ScCIPK17 transiently expressed N. benthamiana challenged by R. solanacearum for 7 days. Figure S7. RT-PCR analysis of ScCIPK21 in the N. benthamiana leaves 2 days after infiltration with Agrobacterium strain GV3101 that carried the vector 35S::00 or 35S::ScCIPKs, in the ScCIPK21 transiently expressed N. benthamiana challenged by R. solanacearum for 1 day, and in the ScCIPK21 transiently expressed N. benthamiana challenged by R. solanacearum for 7 days. [file 12864_2020_7264_MOESM1_ESM.pdf]

New insights into the evolution and functional divergence of the *CIPK* gene family in *Saccharum*

Weihua Su[1,2], Yongjuan Ren[1,2], Dongjiao Wang[1,2], Long Huang[1,2], Xueqin Fu[3], Hui Ling[1], Yachun Su[1,2], Ning Huang[1,2], Hanchen Tang[1,2], Liping Xu[1,2], Youxiong Que[1,2]*

[1]Key Laboratory of Sugarcane Biology and Genetic Breeding, Ministry of Agriculture, Fujian Agriculture and Forestry University, Fuzhou 350002, China

[2]Key Laboratory of Genetics, Breeding and Multiple Utilization of Crops, Ministry of Education, Fujian Agriculture and Forestry University, Fuzhou 350002, China

[3] Key Laboratory of Ministry of Education for Genetics, Breeding and Multiple Utilization of Crops, Plant Immunity Center, Fujian Agriculture and Forestry University, Fuzhou 350002, China.

***Correspondence should be addressed to queyouxiong@126.com**

The full postal address of the submitting author Youxiong Que is as follows: Key Laboratory of Sugarcane Biology and Genetic Breeding, Ministry of Agriculture, Fujian Agriculture and Forestry University, Fuzhou 350002, China

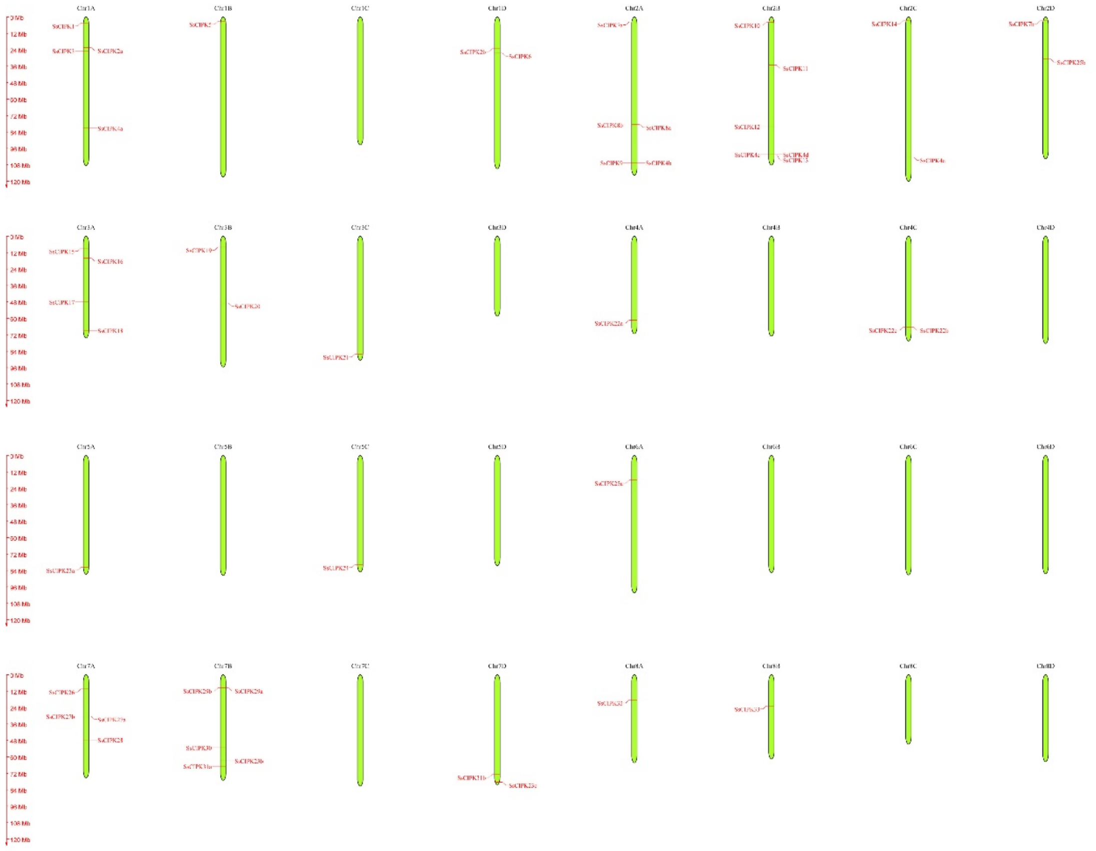

Fig. S1 Chromosomal distribution of *SsCIPKs*. The scale bar on the left indicated the length (Mb) of sugarcane chromosomes.

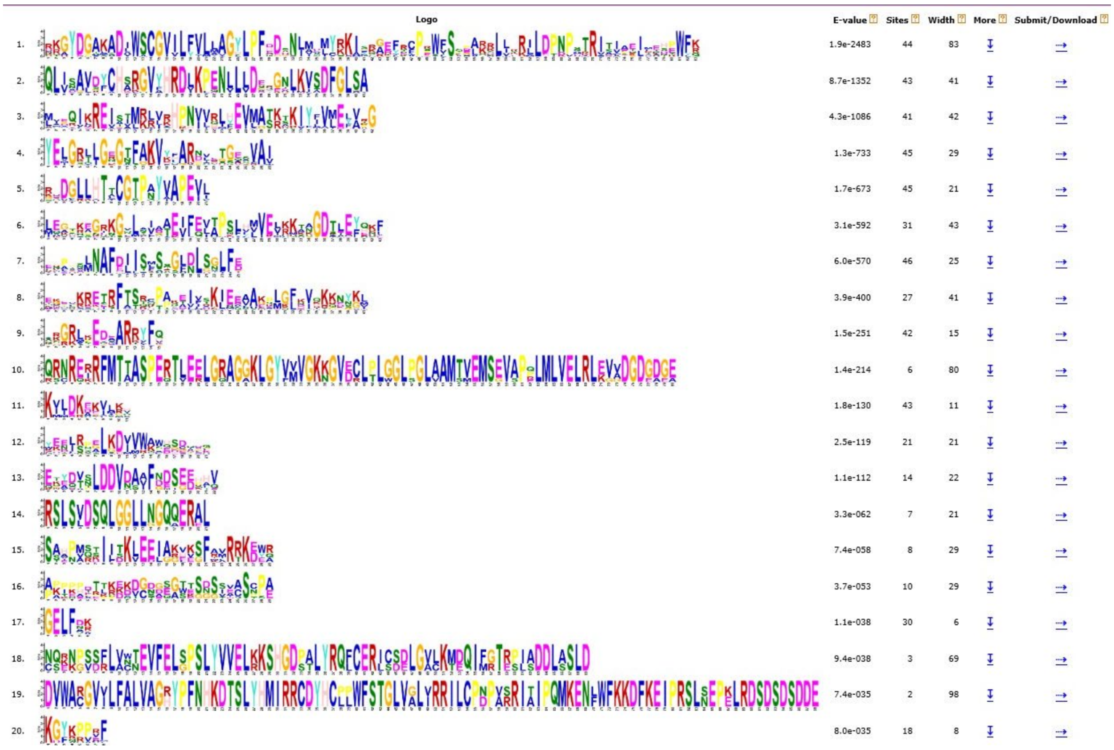

Fig. S2 Analysis and distribution of conserved motifs in SsCIPK proteins.

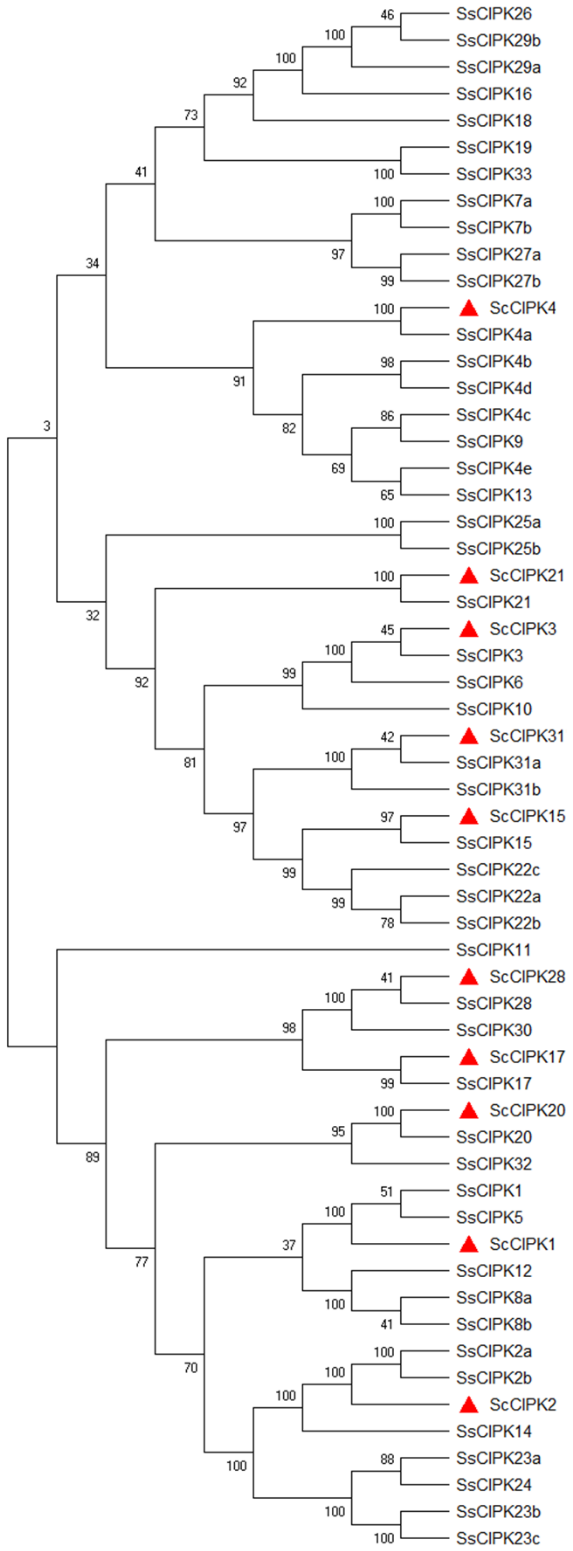

Fig. S3 Phylogenetic analysis of CIPK proteins from *S.spontaneum* and *Saccharum* spp. hybrid (ROC22). Red triangles represent ScCIPK proteins.

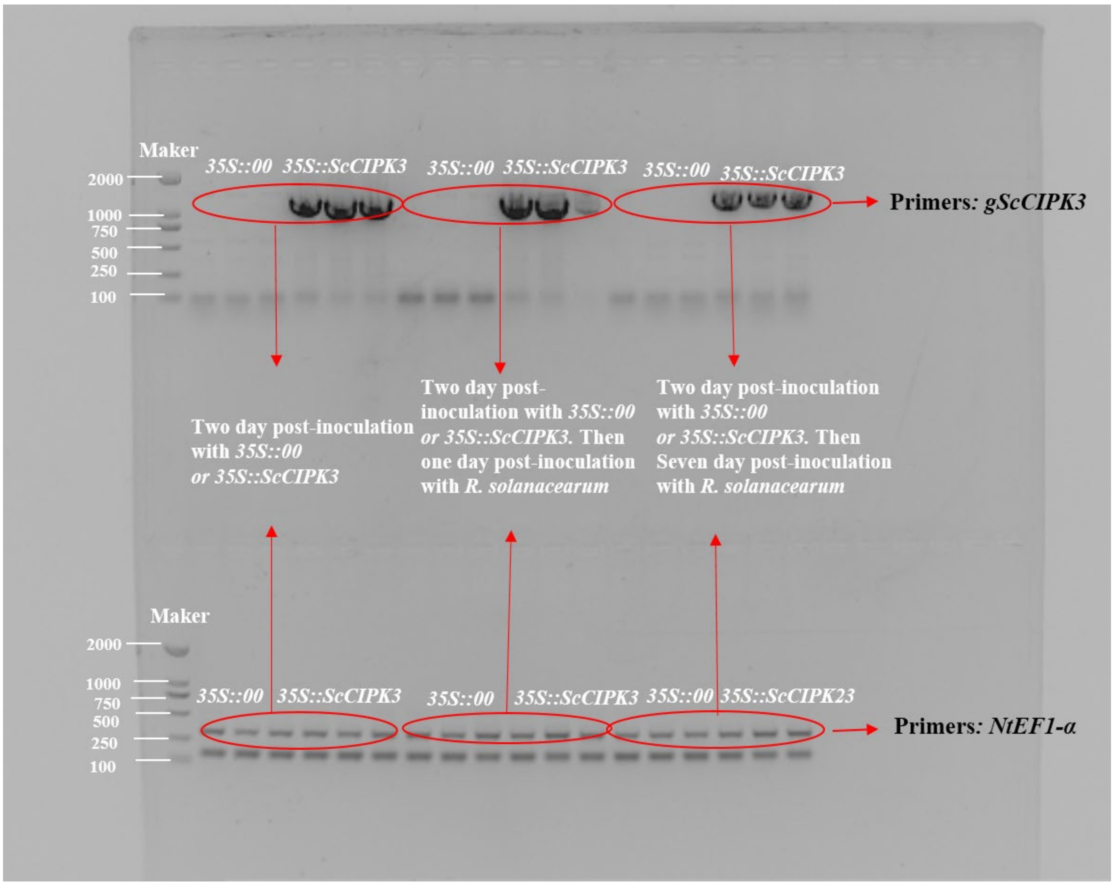

Fig. S4 RT-PCR analysis of *ScCIPK3* in the *N. benthamiana* leaves 2 days after infiltration with *Agrobacterium* strain GV3101 that carried the vector *35S::00* or *35S::ScCIPK3*, in the *ScCIPK3* transiently expressed *N. benthamiana* challenged by *R. solanacearum* for one day, and in the *ScCIPK3* transiently expressed *N. benthamiana* challenged by *R. solanacearum* for seven days.

Note: *35S::00* stands for the control templet, *35S::ScCIPK3* stands for the template of instantaneous expression *ScCIPK3*. *gScCIPK3*and *NtEF1-α* represented the primers used in the RT-PCR.

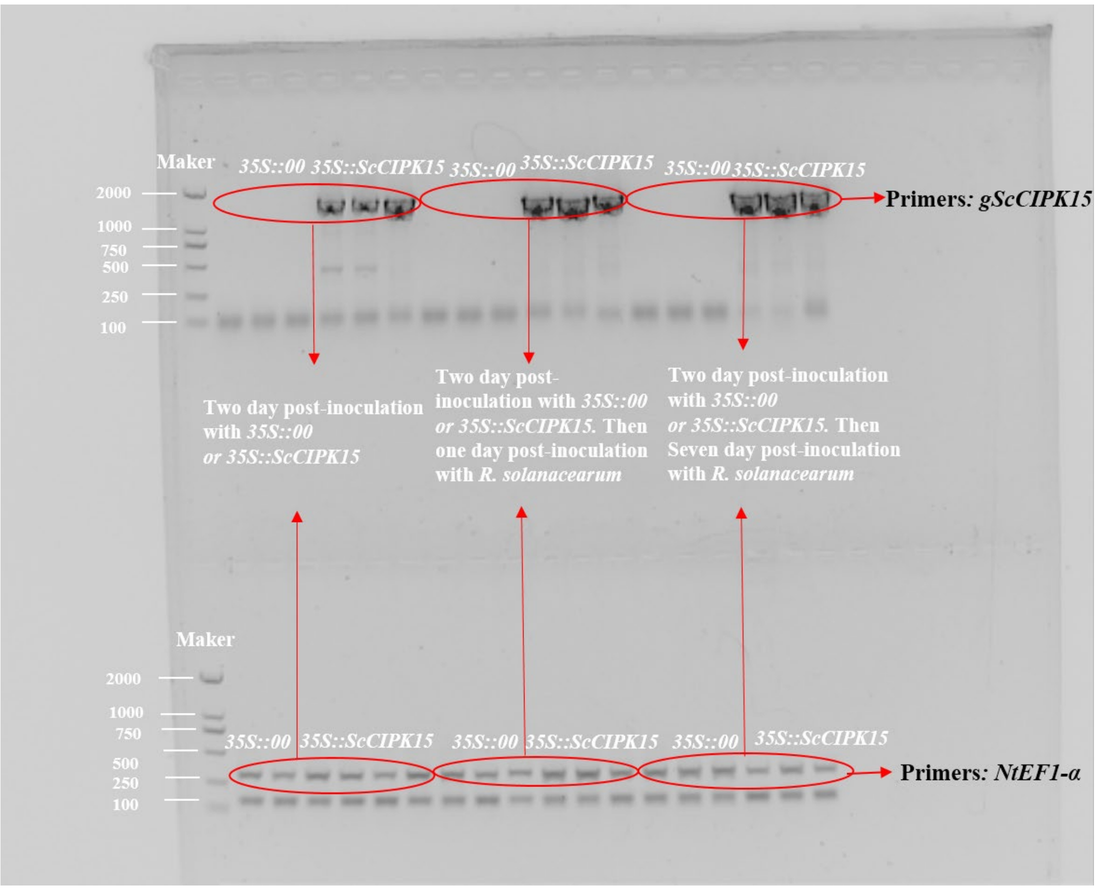

Fig. S5 RT-PCR analysis of *ScCIPK15* in the *N. benthamiana* leaves 2 days after infiltration with *Agrobacterium* strain GV3101 that carried the vector *35S::00* or *35S::ScCIPK15*, in the *ScCIPK15* transiently expressed *N. benthamiana* challenged by *R. solanacearum* for one day, and in the *ScCIPK15* transiently expressed *N. benthamiana* challenged by *R. solanacearum* for seven days. Note: *35S::00* stands for the control templet, *35S::ScCIPK15* stands for the template of instantaneous expression *ScCIPK15*. *gScCIPK15*and *NtEF1-α* represented the primers used in the RT-PCR.

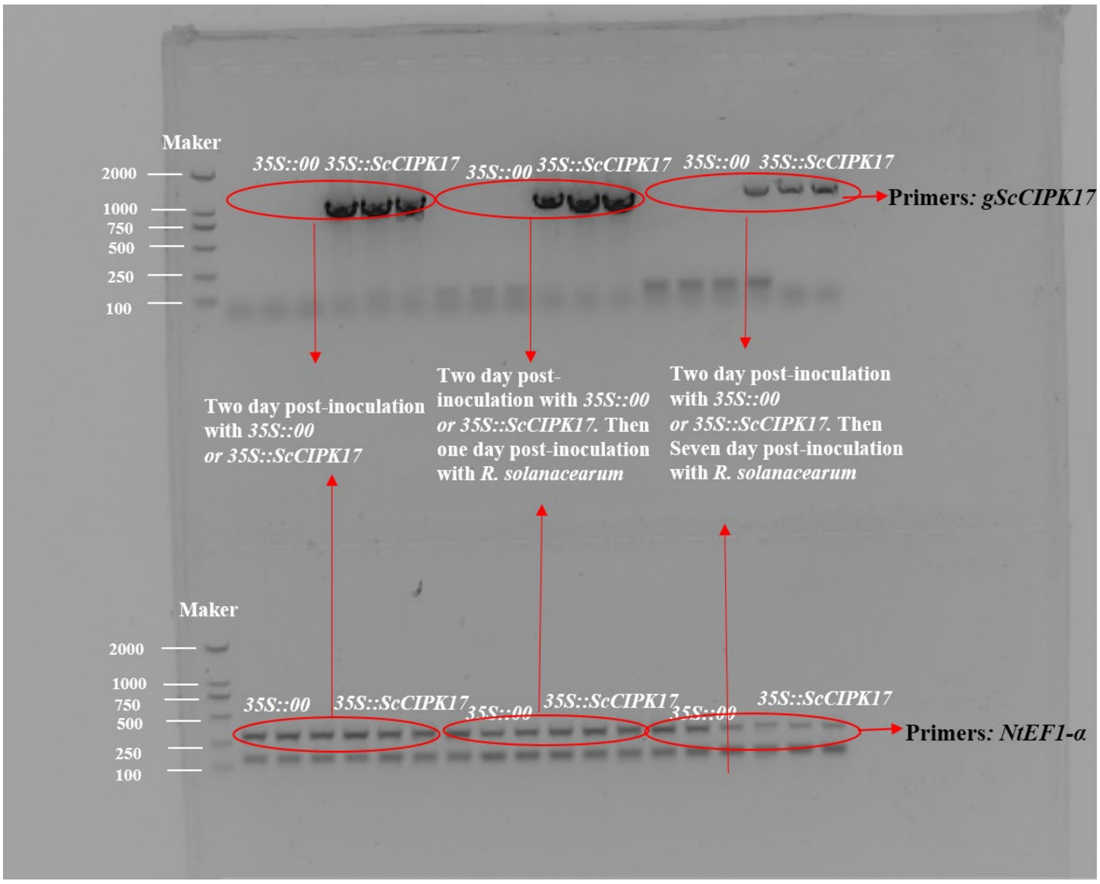

Fig. S6 RT-PCR analysis of *ScCIPK17* in the *N. benthamiana* leaves 2 days after infiltration with *Agrobacterium* strain GV3101 that carried the vector *35S::00* or *35S::ScCIPK17*, in *the ScCIPK17* transiently expressed *N. benthamiana* challenged by *R. solanacearum* for one day, and in *the ScCIPK17* transiently expressed *N. benthamiana* challenged by *R. solanacearum* for seven days. Note: *35S::00* stands for the control templet, *35S::ScCIPK17* stands for the template of instantaneous expression *ScCIPK17*. *gScCIPK17*and *NtEF1-α* represented the primers used in the RT-PCR.

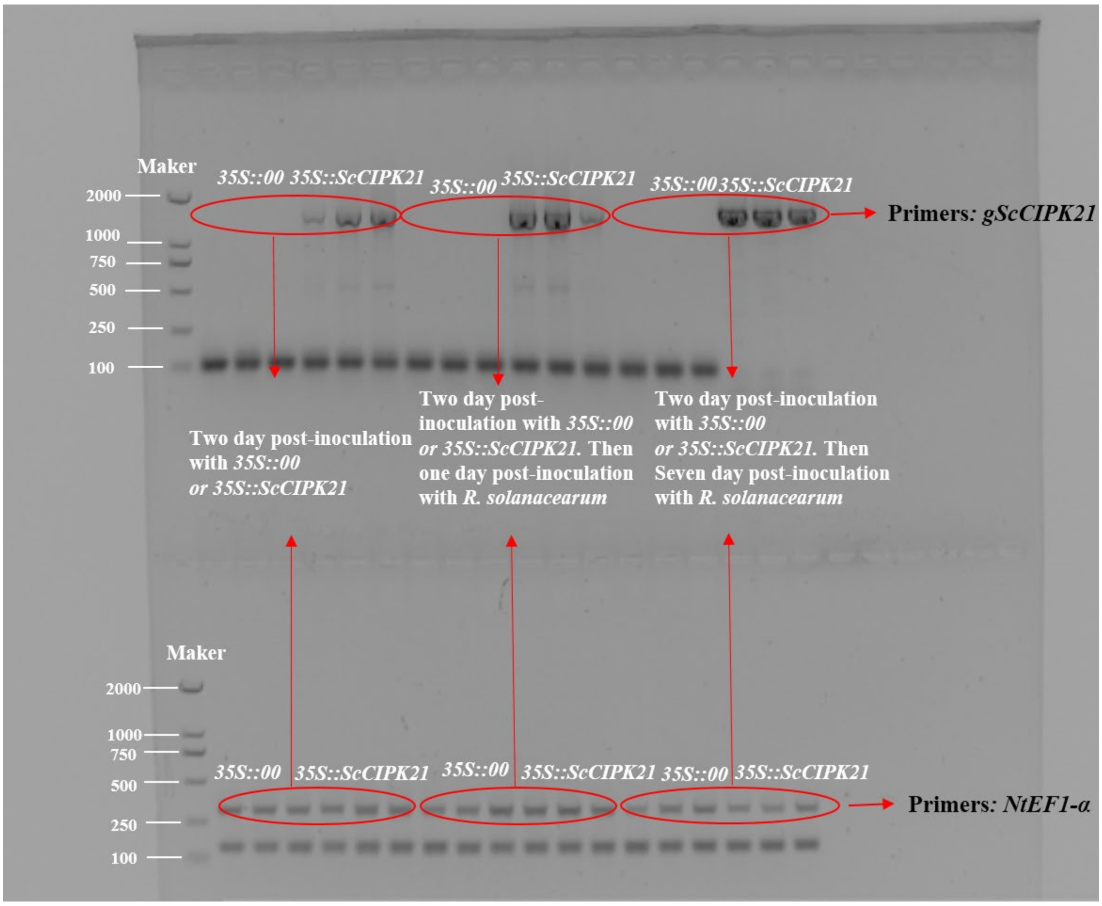

Fig. S7 RT-PCR analysis of *ScCIPK21* in the *N. benthamiana* leaves 2 days after infiltration with *Agrobacterium* strain GV3101 that carried the vector *35S::00* or *35S::ScCIPKs*, in the *ScCIPK21* transiently expressed *N. benthamiana* challenged by *R. solanacearum* for one day, and in the *ScCIPK21* transiently expressed *N. benthamiana* challenged by *R. solanacearum* for seven days. Note: *35S::00* stands for the control templet, *35S::ScCIPK21* stands for the template of instantaneous expression *ScCIPK21*. *gScCIPK21*and *NtEF1-α* represented the primers used in the RT-PCR.
