# Supplementary material for: New insights into the evolution and functional divergence of the CIPK gene family in Saccharum
Source: BMC Genomics. 2020 Dec 7;21:868. doi: 10.1186/s12864-020-07264-9 (PMC7720545; doi:10.1186/s12864-020-07264-9)
Supplement: Supplementary file 6 — Additional file 6: Table S5. Primers used in this study. [file 12864_2020_7264_MOESM6_ESM.pdf]

Table S5 Primers used in this study

Primer	Forward primer (5'-3')	Reverse primer (5'-3')	Strategy
ScCIPK1	AAACCCTTGCCTGTCCGCTTCTTCG	AGGCATCTCACCCTGTGGCAACCAA	Gene cloning
ScCIPK2	CTCGCTTCTCCTTGAGCTCTT	AACCGACTCTGCAATGGACA	Gene cloning
ScCIPK3	CCTTGCTTGGGAATTGGCAG	CAGCGTTACACTTGTCACAATATC	Gene cloning
ScCIPK4	CAAGCCCAGCCCCTGTAAACCACCA	GCAAGAGGAAATGCGATCGAGCAGGAT	Gene cloning
ScCIPK15	TCACCTTGGAGACGACG	ACTGCAGAAATGCCATGTATTCCAG	Gene cloning
ScCIPK17	CACGACCTCTGTCGGCAAT	GTGGCTAATTTCCACCGCTC	Gene cloning
ScCIPK20	AGGCTTGCTCTTCCCGAAATCTCCT	ACTTTGCACAGGGAATCGAGGTACGG	Gene cloning
ScCIPK21	CGCTCGTGAACCATTGTAGC	CGCAATTACAGTCCACCCTCT	Gene cloning
ScCIPK28	TCCCTGCCCTGTATTCCAACCGTCT	TGCGTTGCAGCTGAACTGCTTTGGT	Gene cloning
ScCIPK31	GCATCAATTCCACGCCGTTC	CACCACCATGGTCCTCCAATAC	Gene cloning
qScCIPK1	TAGACCACCTCTTCGGGTGATA	TTGACATCCCATTGCAGTGTTC	qRT-PCR analysis
qScCIPK2	CGCTTGGCTTTGATGTTCAGA	ACAACATCCTTCAGTTGGGTTG	qRT-PCR analysis
qScCIPK3	CGCTCCTCTACCCATCTTTACC	GTTTGGGTATCCTGCCAATTCC	qRT-PCR analysis
qScCIPK4	TGTAAACCACCAAGCCAAAGAG	GAGCTCATACTTGCCGAGGAG	qRT-PCR analysis
qScCIPK15	GCAGTACGTCTGAGGGAAATCA	TTAAGCGTTTGGAGAGCTCCTT	qRT-PCR analysis
qScCIPK17	CTCCCAGGAATCCGTCATCATT	CGCTCAGTGATTCAGTCCTCAT	qRT-PCR analysis
qScCIPK20	AACTACAAAGAGCAGCAGGAGA	ACCCACTCATCAAGTCCATAGT	qRT-PCR analysis
qScCIPK21	AAGGATGTTCAGGTTGCCTTCA	GTCATGAACCGTGAGTTGCTG	qRT-PCR analysis
qScCIPK28	CCCATGGTGATCCTGCATTGTA	AAAGGCGTCGCAGATCGTTT	qRT-PCR analysis
qScCIPK31	CCCAACTTGATGGAGATGTATCG	CTCCTTTACTGTACGGGTCTCC	
CAC	ACAACGTCAGGCAAAGCAAA	AGATCAACTCCACCTCTGCG-3	qRT-PCR analysis
CUL	TGCTGAATGTGTTGAGCAGC	TTGTCGCGCTCCAAGTAGTC	qRT-PCR analysis
gScCIPK3	GGGGACAAGTTTGTACAAAAAAGCAGGCTTC<br>ATGATTGAGAAAGAGAATAT	GGGGACCACTTTGTACAAGAAAGCTGGGTCT<br>TCACCTAGCCAAGCCCACA	Gateway entry vector construction and semi-quantitative analysis
gScCIPK15	GGGGACAAGTTTGTACAAAAAAGCAGGCTTC<br>ATGGATGGTAGGAGGACAAT	GGGGACCACTTTGTACAAGAAAGCTGGGTCG<br>TTGTTTTGTTCCTCTGGAG	Gateway entry vector construction and semi-quantitative analysis
gScCIPK17	GGGGACAAGTTTGTACAAAAAAGCAGGCTTC<br>ATGGTGCTTGAGTTTGTTAA	GGGGACCACTTTGTACAAGAAAGCTGGGTCG<br>AACCCAGATAGAGGGAACG	Gateway entry vector construction and semi-quantitative analysis

gScCIPK21	GGGGACAAGTTTGTACAAAAAAGCAGGCTTC ATGGAGAAGAAGCCGACCAT	GGGGACCACTTTGTACAAGAAAGCTGGGTCA GAGGATGGCTTCAATGGTG	Gateway entry vector construction and semi-quantitative analysis
NtHSR201	CAGCAGTCCTTTGGCGTTGTC	GCTCAGTTTAGCCGCAGTTGTG	qRT-PCR analysis
NtHSR203	TGGCTCAACGATTACGCA	GCACGAAACCTGGATGG	qRT-PCR analysis
NtHSR515	TTGGGCAGAATAGATGGGTA	TTTGGTGAAAGTCTTGGCTC	qRT-PCR analysis
NtPR-1a/c	AACCTTTGACCTGGGACGAC	GCACATCCAACACGAACCGA	qRT-PCR analysis
NtPR2	TGATGCCCTTTTGGATTCTATG	AGTTCCTGCCCCGCTTT	qRT-PCR analysis
NtPR3	CAGGAGGGTATTGCTTTGTTAGG	CGTGGGAAGATGGCTTGTTGTC	qRT-PCR analysis
NtEFE26	CGGACGCTGGTGGCATAAT	CAACAAGAGCTGGTGCTGGATA	qRT-PCR analysis
NtAccdeaminase	TCTGAGGTTACTGATTTGGATTGG	TGGACATGGTGGATAGTTGCT	qRT-PCR analysis
NtEF1-α	TGCTGCTGTAACAAGATGGATGC	GAGATGGGGACAAAGGGGATT	qRT-PCR analysis

Note: attB1 and attB2 adapters were underlined in the primer *gScCIPKs*.
