# Supplementary material for: New insights into the evolution and functional divergence of the CIPK gene family in Saccharum
Source: BMC Genomics. 2020 Dec 7;21:868. doi: 10.1186/s12864-020-07264-9 (PMC7720545; doi:10.1186/s12864-020-07264-9)
Supplement: Supplementary file 7 — Additional file 7: Table S6. The amplification reaction procedures for cloning of sugarcane ScCIPKs. [file 12864_2020_7264_MOESM7_ESM.pdf]

Table S6 The amplification reaction procedures for cloning of sugarcane *ScCIPKs*

Gene name	Pre-denaturation	Denaturation	Annealing	Extension	Cycles	Extension	Enzyme
ScCIPK1	94°C 1 min	98°C 10 s	-	68°C 2 min	30	72°C 10 min	*LA* Taq
ScCIPK2	94°C 4 min	94°C 30 s	55°C 30 s	72°C 2 min	35	72°C 10 min	*Ex* Taq
ScCIPK3	94°C 4 min	94°C 30 s	55°C 30 s	72°C 2 min	35	72°C 10 min	*Ex* Taq
ScCIPK4	94°C 1 min	98°C 10 s	-	68°C 2 min	30	72°C 10 min	*LA* Taq
ScCIPK15	94°C 4 min	94°C 30 s	50°C 30 s	72°C 2 min	35	72°C 10 min	*Ex* Taq
ScCIPK17	94°C 4 min	94°C 30 s	50°C 30 s	72°C 2 min	35	72°C 10 min	*Ex* Taq
ScCIPK20	94°C 1 min	98°C 10 s	-	68°C 2 min	30	72°C 10 min	*LA*Taq
ScCIPK21	94°C 4 min	94°C 30 s	55°C 30 s	72°C 2 min	35	72°C 10 min	*Ex* Taq
ScCIPK28	94°C 4 min	94°C 30 s	45°C 30 s	72°C 2 min	35	72°C 10 min	*Ex* Taq
ScCIPK31	94°C 4 min	94°C 30 s	50°C 30 s	72°C 2 min	35	72°C 10 min	*Ex* Taq
